# A Methanolic Urea-Enhanced Protein Extraction Enabling the Largest Bacterial Phosphorylation Resource

**DOI:** 10.1016/j.mcpro.2025.101019

**Published:** 2025-06-24

**Authors:** Pei-Shan Wu, Ting-An Chen, Bo-Yu Chen, Yasushi Ishihama, Miao-Hsia Lin

**Affiliations:** 1 Department of Microbiology, National Taiwan University College of Medicine, Taipei, Taiwan; 2 Graduate School of Pharmaceutical Sciences, Kyoto University, Kyoto, Japan; 3 Laboratory of Proteomics for Drug Discovery, National Institute of Biomedical Innovation, Health and Nutrition, Ibaraki, Osaka, Japan

**Keywords:** bacterial phosphoproteome, bile challenge, L. monocytogenes, liquid-liquid extraction (LLE), methanolic urea protein extraction (MUPE)

## Abstract

Mass spectrometry (MS)-based phosphoproteomics analysis is a powerful approach for elucidating the regulatory roles of protein phosphorylation across all domains of life. However, bacterial phosphoproteomics still faces significant technical challenges due to the extremely low substoichiometry of phosphorylation evens and the structural complexity of bacterial cell envelopes, which impede efficient cell lysis, protein recovery, and purity. To address these obstacles, we developed Methanolic Urea-enhanced Protein Extraction (MUPE), a streamlined, detergent-free, solvent-based method that leverages the amphiphilic nature of methanol and the chaotropic properties of urea to enhance protein yield and lysis efficiency. Furthermore, MUPE seamlessly integrates with liquid–liquid extraction, enabling efficient protein purification without requiring sample transfer and complex manipulations. This workflow significantly improves phosphoproteome coverage and quantitative accuracy across Gram-positive and Gram-negative bacteria while minimizing sample input requirements. Our datasets substantially expand the known landscape of bacterial *O*-phosphorylation, revealing distinct phosphorylation preferences within bacterial signaling networks. Application of MUPE to *Listeria monocytogenes* under bile insult revealed extensive phosphorylation changes independent of protein expression, highlighting phosphorylation as a rapid and dynamic regulatory mechanism. Collectively, MUPE provides a robust and scalable platform for bacterial phosphoproteomic studies, advancing our understanding of phosphosignaling in the context of bacterial physiology and pathogenesis.

## Highlights

• MUPE streamlines protein extraction, cleanup, and phosphopeptide enrichment.
• MUPE shows superior phosphoproteome coverage and reproducibility across bacteria.
• MUPE suits limited samples and multi-omics integration.
• Expanded bacterial *O*-phosphoproteome atlas reveal distinct phosphorylation patterns.
• *O*-phosphorylation acts as a sensing mechanism to trigger stress adaption in bacteria.

## Graphical Abstract

**Figure:**
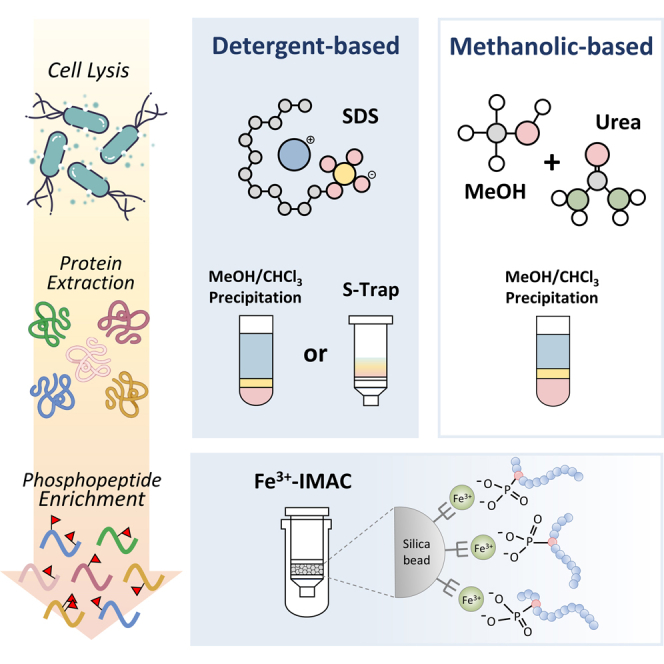

## In Brief

Bacterial phosphoproteomics face challenges from extremely low phosphorylation stoichiometry and complex cell envelopes. We developed Methanolic Urea-enhanced Protein Extraction (MUPE), a detergent-free, solvent-based approach that significantly enhances lysis efficiency, protein yield, and phosphoproteome coverage across diverse bacterial species, with exceptional quantitative accuracy. Our datasets, comprising ∼21,000 *O*-phosphosites, uncover unique phosphorylation motifs and substantially expand the bacterial *O*-phosphorylation landscape. This streamlined, broadly-applicable method enables comprehensive analysis of phosphorylation-mediated signaling in bacterial physiology and pathogenesis, offering insights for novel antimicrobial strategies.

Protein phosphorylation is essential for regulating a wide range of physiological functions across all kingdoms of life, serving as a critical mechanism for controlling various cellular processes. For years, studies of protein phosphorylation in bacteria have predominantly focused on histidine/aspartate (His/Asp) phosphorylation, known for its role in two-component signaling and phosphotransferase systems (1). However, recent research has demonstrated that serine/threonine/tyrosine (Ser/Thr/Tyr) phosphorylation, also known as *O*-phosphorylation, is crucial for bacterial physiology and pathogenesis, a role once thought exclusive to eukaryotes (2). It is now well-awareness that Ser/Thr/Tyr kinases and phosphatases, widespread in bacteria, orchestrate complex regulatory networks involved in host infections, antibiotics resistance, bacterial survival and morphological changes (2, 3, 4, 5). Consequently, characterizing bacterial *O*-phosphorylation dynamics at a system-wide level is paramount in advancing our knowledge of bacterial biology and pathogenicity, highlighting its a promising target for novel antibacterial therapies and a key focus in microbiological research.

Currently, mass spectrometry (MS)-based phosphoproteomic analysis is an exceptional technique for systematically determining protein *O*-phosphorylation dynamics. Despite significant advancements in site-specific phosphorylation profiling, bacterial phosphoproteomics remains relatively unsophisticated (6, 7). Challenges stem from the rigidity of the cell wall, extremely low substoichiometry (approximately 80 times less than in eukaryotes) (8), and interference from abundant substances like phospholipids, peptidoglycans, and lipopolysaccharides. Therefore, bacterial phosphoproteomics often requires a large sample size to meet the MS detection limit for comprehensive profiling (2) and complicated sample handlings. In 2007, Mann group presented the first MS-based bacterial phosphoproteome in *Bacillus subtilis*, utilizing a combination of strong cation exchange and metal oxide chromatography with a 10 mg protein input (9). This method has been standardized for enriching bacterial phosphopeptides, yielding approximately 100 phosphosites from 10 to 30 mg proteins for each species (2, 10, 11, 12). Meanwhile, stepwise hydroxy acid-modified metal oxide chromatography (HAMMOC) for phosphopeptide enrichment has been introduced in *Rhodopseudomonas palustris*, achieving comparable phosphoproteome coverage (13). In 2015, Lin *et al*. achieved ten times higher phosphoproteome coverage by integrating impurity removal with single HAMMOC enrichment, enabling the identification of bacterial-specific phosphorylation features (14). The Heck group improved ionization efficiency and phosphopeptide identification by including nuclease treatment before protein precipitation and phosphopeptide enrichment (15, 16). Nevertheless, sample preparation continues to pose a significant challenge in achieving comprehensive and reproducible bacterial phosphoproteomics, with no optimal solution yet established.

The varied structural complexity and lysis-resistance of bacterial cell envelope necessitate robust cell disruption, typically achieved by combining chemical agents, such as detergents or chaotropic agents (e.g., SDS and urea), with physical or mechanical disruption methods, including heat, ultrasonication, or grinding (17, 18). However, these reagents often interfere with enzymatic digestion and LC-MS analysis, requiring additional steps for contaminant removal and protein purification. Recently, protein aggregation approaches, whether organic solvent-induced or acid-assisted, have gained popularity for extracting proteins in proteomic sample preparation. Techniques like single-pot, solid-phase-enhanced sample preparation (SP3), solvent precipitation SP3 (SP4), and protein aggregation capture (PAC) are now considered gold standards for contaminant removal (19, 20, 21, 22). On the other hand, acid-based protein extraction methods, such as suspension trapping (S-Trap) and sample preparation by easy extraction and digestion (SPEED), have demonstrated superior performance in proteomic analysis across various sample types (23, 24, 25). However, these approaches often involve extensive sample transfer and handling steps, and require specialized materials or equipment, which may lead to sample losses, bias, and potential contamination, and high costs. Such limitations make MS-based phosphoproteomic analysis labor-intensive and less practical.

Organic solvents have long been employed for direct cell lysis with liquid-liquid extraction (LLE) in metabolomics workflows, utilizing a simple partition strategy, such as methanol/chloroform (MeOH/CHCl_3_), to separate target molecules from unwanted substances, typically proteins, (26, 27, 28). The addition of ion salts to organic solvents can modulate protein partitioning, thereby enhancing extraction efficiency to varying degrees (29). Among organic solvents, MeOH stands out for its amphiphilic characteristics, providing strong solubility for protein and effective interactions with diverse protein structures. At high concentrations, MeOH disrupts bacterial cell walls by perturbing membrane fluidity and permeability, facilitating efficient cell lysis (30, 31). These properties suggest that MeOH-based organic solvent lysis and partitioning can be fine-tuned to improve bacterial protein extraction and purification, offering a promising approach for bacterial phosphoproteomics studies, where protein extraction efficiency is critical (14, 15).

In this study, we introduce a rapid, detergent-free, and high-performance protocol for universal bacterial phosphoproteomic analysis, termed as methanolic urea protein extraction (MUPE). This single-tube workflow integrates MeOH- and urea-assisted cell lysis with MeOH/CHCl_3_-based protein precipitation to enable efficient cell disruption, protein extraction, and contaminant removal, while minimizing sample loss, solvent consumption, and manual handling. Compared to conventional SDS-based workflows, MUPE provides a more effective extraction strategy for downstream phosphopeptide enrichment, improving the identification and quantification of bacterial phosphoproteomes. Of noted, the inclusion of urea significantly enhances cell lysis, protein solubility, and dissociation from contaminants, thereby increasing protein recovery. Applying MUPE to both Gram-positive and Gram-negative bacteria resulted in the most comprehensive atlas of bacterial *O*-phosphorylation, revealing distinct phosphorylation preferences across species. Furthermore, the robustness and consistency of MUPE enabled the detection of rapid *O*-phosphorylation signaling events that precede protein expression changes during bile stress responses in *Listeria monocytogenes*, highlighting its utility for studying dynamic phosphosignaling.

## Experimental Procedures

### Chemicals and Reagents

Sodium dodecyl sulfate (SDS), tris(2-caboxyethyl)phosphine hydrochloride (TCEP), 2-chloroacetamide (CAA), triethylammonium bicarbonate (TEABC), trifluoroacetic acid (TFA), sodium chloride (NaCl), iron (III) chloride (FeCl_3_), ammonium phosphate (NH_4_H_2_PO_4_), formic acid (FA), acetic acid (AA), chloroform (CHCl_3_), methanol (MeOH), acetonitrile (ACN), porcine bile extract, and phosphatase inhibitor cocktail 2/3 were purchased from Sigma-Aldrich. Protease inhibitor cocktail (EDTA-Free) was purchased from BIOTOOL. Ethylenediaminetetraacetic acid (EDTA) and urea was obtained from Merck Millipore. Pierce bicinchoninic acid (BCA) protein assay kit was purchased from Thermo Fisher Scientific. MS-grade Lys-C (lysl endopeptidase) and modified sequencing-grade trypsin were purchased from Wako and Promega, respectively. C18 and SDB-XC (poly(styrenedivinylbenzene) copolymer) Empore disks were obtained from 3M and VWR (Randor, PA, USA), respectively. Nickel nitrilo-triacetic acid (Ni-NTA) agarose beads and C18 beads (ODS-AQ-HG, 120 Å, 50um) were purchased from QIAGEN (Hilden, Germany) and YMC, respectively. For bacterial culture, DifcoTM LB (Luria Bertani) Broth Miller and Brain heart infusion (BHI) broth were obtained from BD Biosciences and Biolife, respectively.

### Bacterial Strains and Culturing

*L. monocytogenes* (ATCC 19117, *L. monocytogenes*), *B. subtilis* strain 168 (ATCC23857, *B. subtilis*), and *Staphylococcus aureus* Newman strain (ATCC25904, *S. aureus*) were used as Gram-positive bacteria models in this study; for Gram-negative models, *Escherichia coli* strain K-12 (BW25113, *E. coli*) and *Pseudomonas aeruginosa* reference strain PAO1 (ATCC47085, *P. aeruginosa*) were employed. *S. aureus*, *B. subtilis*, *E. coli* and *P. aeruginosa* were grown in LB medium with vigorous shaking at 37 °C. *L. monocytogenes* was grown in BHI broth with the same culture condition. Cells were collected by centrifugation at stationary phase (OD_600_ = 1.2 for glucose-fed cells) and washed three times with ice-cold PBS. Cell pellet was collected as equal amount per tube (10 mg for *L. monocytogenes*, *E. coli* and *P. aeruginosa*; 7 mg for *B. subtilis*; 50 mg for *S. aureus*) and stored at −80 °C until further experimental processes.

### Standard Protein Extraction Workflow

For SDS workflow, bacterial pellet was lysed with SDS lysis buffer (5% SDS, 100 mM Tris-HCl (pH 9.0), protease inhibitor and phosphatase inhibitors) at the ratio of bacterial pellet: lysis buffer = 10 mg: 400 μl (w/v). Sample was heated at 95 °C for 5 min, homogenized using Bioruptor Plus at 4 °C with 40 and 25 cycles for Gram-positive and Gram-negative bacteria, respectively (30s on/30s off per cycle, high energy mode), and centrifuged at 17,000*g* for 30 min under 4 °C to remove cell debris. The supernatant was subjected for protein precipitation by mixing with 4-fold sample volume of MeOH, 1-fold sample volume of CHCl_3_, and 3-fold sample volume of H_2_O. After centrifugation at 17,000*g* for 10 min under RT, the upper layer was removed without disturbing the interface and three volumes of MeOH were added. After vortexing, protein precipitate was collected by centrifugation at 17,000*g* for 10 min at RT and the white protein precipitate was allowed to air dry and dissolved with 8 M urea in 50 mM TEABC. For strategy comparison, 8 M urea in 50 mM TEABC contained with and without 5% SDS were used as lysis buffers for SU and UA procedure, respectively. To avoid carbamylation caused by urea, the SU and UA samples were not heated to 95 °C. Subsequent protein purification steps were performed as outlined in the procedure above.

### Methanolic Protein Extraction Workflow

For ME workflow, bacterial pellet was well-resuspended and lysed in pre-cold 50% MeOH (10 mg pellet/400 μl buffer). Sample mixture was homogenized with Bioruptor at 4 °C with 40 and 25 cycles for Gram-positive and Gram-negative bacteria, respectively (30s on/30s off per cycle, high energy mode), then subjected for protein precipitation by sequentially adding 1/2-fold sample volume of MeOH and CHCl_3_ (final ratio of MeOH: CHCl_3_: H_2_O = 2: 1: 1, v/v/v) and mixed vigorously. After standing on-ice for 30 min, protein precipitate was collected by centrifugation at 17,000*g* for 10 min under 4 °C, and dissolved with 8 M urea in 50 mM TEABC.

To test the efficiency of ion salts combined 50% MeOH as a lysis buffer, different constitution of ion salts (ME’), including 50 mM and 100 mM Tris-HCl, and 1 M, 2 M, 4 M, 6 M urea, were added into 50% MeOH. Tris-HCl in 50% MeOH was prepared from 1 M Tris-HCl (pH 9.0) buffer diluted with H_2_O and MeOH into the final concentration of 50 mM and 100 mM; urea in 50% MeOH was prepared by directly dissolving different weights of urea powder in 50% MeOH to obtain the final urea concentrations close to 1 M, 2 M, 4 M and 6 M. Following, sample was homogenized with Bioruptor and protein was precipitated by adding 1/2-fold sample volume of MeOH, CHCl_3_ and H_2_O (final ratio of MeOH: CHCl_3_: H_2_O = 2: 1: 2, v/v/v), then vortexed vigorously. Protein pellet was collected by centrifugation at RT and dissolved using 8 M urea in 50 mM TEABC.

### In-Solution Digestion

In-solution digestion was carried out for standard and methanolic protein extraction workflows. Total proteins extracted from equal weights of bacterial pellets using each method were reduced and alkylated at 29 °C by 10 mM TCEP for 30 min and 40 mM CAA for 45 min, respectively. Protein mixture was diluted with 50 mM TEABC to a urea concentration of 4M, followed by digestion with Lys-C (1:100, w/w) for 3 h at 29 °C. The mixture was then further diluted to a urea concentration of 2 M and digested with trypsin (1:50, w/w) for 16 to 20 h at 29 °C. The digested peptide sample was acidified with TFA and desalted with a homemade StageTip packaged with SDB-XC membrane and C18-beads. The concentration of protein and peptide were measured by BCA assay.

### Protein Extraction and S-Trap Digestion

*L. monocytogenes* samples prepared using S-Trap micro spin column followed the manufacturer’s guideline, with 5% SDS in 50 mM TEABC as lysis buffer, and the samples were heated at 95 °C for 5 min. Additionally, a modified method using 5% SDS in 50 mM TEABC containing 8 M urea (25) for cell lysis was also applied to the bacterial phosphoproteome, without heating at 95 °C. The bacterial pellet to lysis buffer ratio was 10 mg of pellet per 400 μl of buffer. Samples were thoroughly mixed and homogenized by Bioruptor (40 cycles, 30 s on/30 s off per cycle, high energy mode) at 4 °C. The protein extracts were then collected by centrifugation at 17,000*g* for 30 min under 4 °C. The entire protein extracts were reduced with 10 mM TCEP for 30 min and alkylated with 40 mM CAA for 45 min at 29 °C. The alkylated sample was acidified by adding TFA to a final concentration of 10% and mixed with six volumes of binding buffer (90% MeOH; 100 mM TEABC). After gentle vertexing, the protein mixture was loaded onto S-Trap micro spin column (ST, 100 μg protein/column) and centrifuged at 4000g for 2 to 3 min. Then sample was washed with binding buffer for three times and digested with Lys-C and trypsin at a protein-to-enzyme ratio of 100: 1 (w/w) under 47 °C for 3 h. Tryptic peptides were eluted with 80% ACN contained 1% TFA by centrifuging at 2000*g* for 3 min and desalted with StageTip packaged with C18 beads.

### Phosphopeptide Enrichment

Five percent of the peptide sample was allocated for proteome analysis, while the remaining 95% was used for phosphopeptide enrichment. The phosphopeptides were enriched by immobilized metal affinity chromatography (IMAC) according to previous work (32). Briefly, Ni-NTA agarose beads were suspended with 6% AA (pH 3.0) and loaded into a D200 micro-tip with a frit-disk capped in the end, then deactivated with 50 mM EDTA in 1M NaCl. The beads were equilibrated with 6% AA, activated with 100 mM FeCl_3_ in 6% AA and equilibrated with 6% AA again. The peptide concentration was adjusted to ∼1 μg/μl in 80% ACN with 0.1% TFA. Subsequently, the peptide mixture was loaded onto an activated micro-tip and washed twice with 80% ACN containing 0.1% TFA and 1% AA. The phosphopeptides were eluted with 200 mM NH_4_H_2_PO_4_ and desalted with homemade D200 StageTip packed with two-layer C18 membrane. Phosphopeptide sample was dried with vacuum centrifugation and dissolved in 0.1% FA before LC-MS/MS analysis.

### Bile Treatment in *L. monocytogenes*

Cultures were grown in BHI broth at 37 °C to mid-log phase. The cells were centrifuged at 1000*g* for 10 min and treated with bile acids by resuspending bacteria in BHI broth contained 1% porcine bile acids, the corresponding control was resuspended in normal BHI broth. After 30 min treatment at 37 °C, cells were immediately collected by centrifugation and lysed (10 mg of bacterial pellet) with 50% MeOH containing 4 M urea. Protein extraction was followed MUPE workflow described above, following protein extract was subjected for tryptic digestion and phosphopeptide enrichment.

### LC-MS/MS Analysis

Peptide and phosphopeptide samples were analyzed with Orbitrap Fusion Lumos mass spectrometer coupled with UltiMate 3000 RSLCnano system (Thermo Fisher Scientific). Sample mixture was loaded onto a nanoEase M/Z Peptide CSH C18 column (130 Å, 1.7 μm, 75 μm × 250 mm; Waters). The peptide sample was separated through a gradient from 1.6% to 38.5% buffer B (100% ACN with 0.1% FA) over 80 min, and the phosphopeptide was separated by a gradient from 1.6% to 50% buffer B over 100 min. The flow rate was set at 300 nl/min and the column temperature was set at 45 °C. Buffer A was 0.1% FA in water. The mass spectrometer was operated in data-dependent acquisition (DDA) with Top-Speed mode for a cycle time as 3 s. Survey full scan MS spectra were acquired in the orbitrap with mass range of *m/z* 350 to 1500 for proteome and *m/z* 400 to 1250 for phosphoproteome. The resolution was set at 60 K, automatic gain control (AGC) target at 4e5 and maximum injection time (IT) at 50 ms. The most intense ions were sequentially selected for HCD MS/MS fragmentation with a normalized collision energy (NCE) of 30% and recorded at resolution of 30 K and 15 K for proteome and phosphoproteome, respectively. The isolation window was set at 1.4 Th with AGC target of 5e4. The maximum IT was set at 54 ms for proteome and 75 ms for phosphoproteome. Advanced peak detection function is on with a precursor fit threshold of 70% at 1.4 *m/z* window. The precursor ions with 2^+^ to 7^+^ charges were selected for HCD fragmentation, and exclusion duration was set as 20 s.

### Data Processing

The raw MS/MS data were analyzed with Proteome Discoverer (PD, version 2.4.1.15, Thermo Fisher Scientific) by SequestHT engine against NCBI RefSeq databases of *L. monocytogenes* ATCC 19117 (download on 2023.01, 2868 protein entries), Swissprot databases of *B. subtilis* strain 168 (download on 2023.12, 4226 protein entries), *E. coli* strain K12 (download on 2022.12, 4634 protein entries) and *Pseudomonas saeruginosa* (download on 2020.07, 3784 protein entries), UniProtKB TrEMBL database of *S. aureus* tax426430 (download on 2023.01, 3674 protein entries), and common contaminants for peptide and protein identification. The search parameters were set as follows: mass tolerance was set at 10 ppm for precursor and 0.05 Da for fragment, trypsin specificity allowing up to two missed cleavages, carbamidomethyl (C, +57.021 Da) was set as fixed modification; oxidation (M, +15.995 Da), acetylation (protein N-term, +42.011 Da), and phosphorylation (STY, +79.966 Da, for phosphoproteome) were set as dynamic modifications. The minimal peptide length was seven residues. False discovery rate (FDR) of peptide and protein were both set at 1%. Phosphosites with a localization probability of ≥0.75 were used for further analysis. Quantitative proteins and phosphopeptides were further analyzed by Perseus (v 1.6.15.0). For comparison of bile acid treatment in *L. monocytogenes*, protein and phosphopeptide abundance were transformed into log2 scale, then the imputation of missing values was performed for phosphopeptides with at least two valid values per condition (three technical replicates).

### Experimental Design and Statistical Rationale

All of MS datasets were performed with three technical or biological replicates in each experimental condition. All values were presented as mean ± standard deviation (SD). The differential significance between sample groups was calculated using two sample *t* test and the threshold was set as *p*-value <0.05 without statistical correction. Data visualization was carried out by using Graphpad Prism software, Microsoft Excel and R program. GRAVY index was performed by using GRAVY Calculator (https://www.gravy-calculator.de/) and the calculated isoelectric point (pI) values were extracted from PD search results. KEGG pathway enrichment and cellular component annotation were done by using BlastKOALA (33) and Gene Ontology (GO), respectively. For motif sequence analysis, sequence window surrounds the phosphosite (±7) obtained from PD search result was submitted to pLogo generator (v1.2.0) (34) for potential motifs analysis, utilizing protein sequences in search FASTA files as backgrounds.

## Results

### Enhanced Phosphoproteomic Coverage via Urea-Assisted Solvent Extraction

**Fig. 1:**
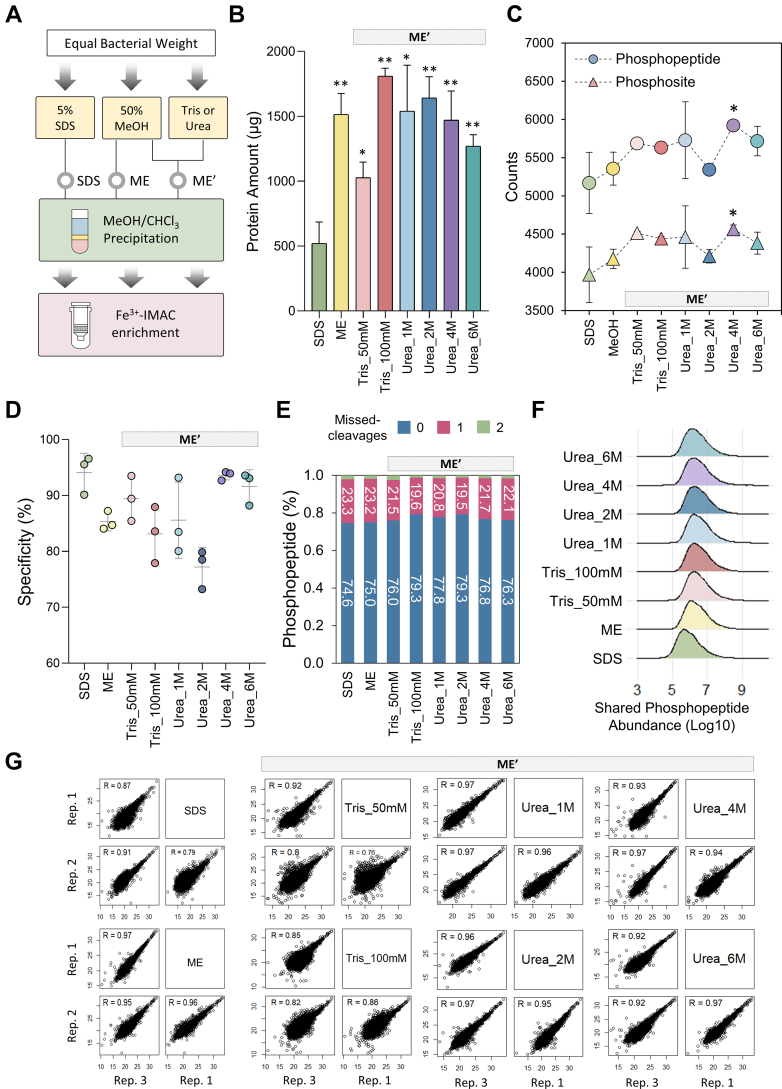
**Comparison of phosphoproteome performance for ME and ME‘ protein extraction strategies with SDS as the conventional control in *L. monocytogenes*.***A*, brief illustration of the control SDS method and the ME and ME’ workflows. *B*, bar chart represented the protein yield from equal bacterial pellet weights. *C*, the identified numbers of unique phosphopeptides (○) and class I phosphosites (▵) across different methods. *D*, dot plot indicated the phosphopeptide enrichment specificity across different methods. *E*, cumulative bars showed the percentage of missed cleavages for identified phosphopeptides. The value labeled in the center means the percentage. *F*, violin plot depicted the distribution of log10-transformed abundance of commonly identified phosphopeptides across all sample groups in technical triplicate (3 valid values). *Red dashed line* in the *middle* represented the median. *G*, scatter plots showed the technical reproducibility with the Pearson’s correlation coefficient marked on *upper left*. All data are presented with mean ± SD (*N* = 3), and significance was determined using two sample *t* test (∗*p*-value <0.05). SDS served as the comparative control for statistical analysis. ME, the methanol-alone cell lysis workflow; ME’, the workflow with the addition of different concentration of Tris or urea to MeOH for cell lysis, including 50/100 mM Tris and 1/2/4/6 M urea; SDS, the conventional SDS-based lysis workflow.

MS-based phosphoproteomics is a powerful tool for accurately mapping phosphosite dynamics, involving protein extraction, digestion, peptide desalting, and phosphopeptide enrichment. Amongst, effective protein recovery and impurity removal are essential for achieving comprehensive phosphoproteome coverage while preserving the physiological state of the sample. In this study, we developed a streamlined, detergent-free workflow for bacterial phosphoproteome analysis, using *L. monocytogenes* as the model organism. Organic solvents like MeOH, ACN, and isopropanol, are widely used in metabolomics for direct cell lysis and metabolites isolation (26, 27). However, their potential as detergent alternatives in proteomics, particularly for challenging samples like tissues or Gram-positive bacteria, remains underexplored. Moreover, their compatibility with phosphoproteomic analysis requires systematical evaluation to ensure effective protein extraction and purification, as well as suitability for downstream phosphopeptide enrichment, factors that are critical for achieving comprehensive bacterial phosphoproteome analysis. As illustrated in Figure 1*A*, equal weights of cell pellets were lysed using 50% MeOH (ME), 50% MeOH supplemented with varying concentrations of Tris or urea (ME’), or 5% SDS in 100 mM Tris (SDS) as a control. Following ultrasonication-assisted cell disruption, lysates were directly subjected to MeOH/CHCl_3_ protein precipitation, trypsin digestion, and Fe^3+^-IMAC phosphopeptide enrichment. Here, Tris was included at concentration of 50 mM and 100 mM to stabilize pH and prevent protein denaturation, while urea was tested at 1 M, 2 M, 4 M, and 6 M to enhance cell disruption, promote protein solubilization, and solvent-protein partitioning efficiency (35, 36).

With equal amounts of *L. monocytogenes* biomass, both ME and ME’ yielded approximately twice the proteins and peptides quantities, and identified more phosphopeptides and class I phosphosites compared to SDS-based methods (Fig. 1, *B* and *C*, and Supplemental Tables S1 and S2). The ME’ workflow, with 50% MeOH supplemented with 4 M urea, showed the most significant improvement, identifying 7006 phosphopeptides and 5366 phosphosites, approximately 1000 more phosphopeptides and 600 more phosphosites than the SDS approach (Fig. 1*C*, Supplemental Table S2, *A* and *G*). All tested methods achieved phosphopeptide enrichment efficiency above 70%, with SDS and ME’ (4 M or 6 M urea) exceeding 90% (Fig. 1*D*). Missed-cleavage rates and the distribution of phosphoacceptors (Ser/Thr/Tyr), as well as the proportions of singly, doubly, and multiply phosphorylated peptides, were similar across SDS, ME, and ME’, with ME’ exhibiting slightly higher digestion efficiency (Fig. 1*E* and Supplemental Fig. S1, *A* and *B*). These observations indicate that differences primarily stem from protein recovery and sample loss rather than phosphosite characteristics. Although most workflows identified ≥4000 phosphosites, only ∼27% were shared across all conditions (Supplemental Fig. S1*C*), reflecting the wide dynamic range of the phosphoproteome, stochastic nature of MS analysis, and the significant impact of sample preparation on coverage depth (6, 7). Commonly identified phosphopeptides showed higher median abundances in ME and ME’ conditions, suggesting superior impurity removal by MeOH-based methods (Fig. 1*F*). Furthermore, ME and ME’ with urea demonstrated high reproducibility, with Pearson's correlation coefficients of 0.92 − 0.97 across replicates (Fig. 1*G*).

These results demonstrate that cell lysis using 50% MeOH supplemented with 4 M urea, followed by MeOH/CHCl_3_ protein precipitation, markedly enhances protein extraction efficiency and contaminant removal. This protocol is fully compatible with Fe^3+^-IMAC enrichment and improved identification depth and quantitative accuracy in bacterial phosphoproteomics. We designate this method Methanolic Urea-enhanced Protein Extraction (MUPE), underscoring its critical role in effective cell lysis and protein recovery to advance phosphoproteomic analysis.

### MUPE-Enabled Comprehensive Coverage and High Reproducibility in Bacterial Phosphoproteome Analysis

**Fig. 2:**
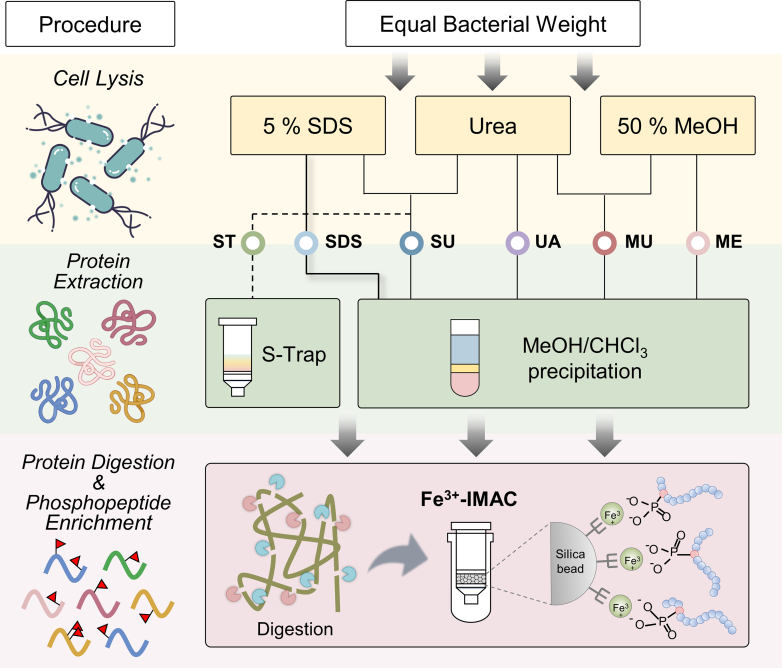
**Brief illustration of different protein extraction workflow for bacterial phosphoproteome analysis.** Equal cell pellet (10 mg) amount was used as starting material for protein extraction. Cells were suspended in lysis buffers containing 5% SDS with (SU and ST) or without (SDS) urea, 50% MeOH with (MU) or without (ME) urea, or 8 M urea (UA). Protein purification was performed using either MeOH/CHCl_3_ precipitation for SDS, SU, ME, MU, and UA or S-trap filtration for ST. The digested peptide was desalted for Fe^3+^-IMAC phosphopeptide enrichment.

To our knowledge, a comprehensive survey of sample preparation for bacterial phosphoproteome analysis remains absent. Given the increasing use of protein aggregation-based methods for interference removal, we benchmarked our MUPE workflow by further incorporating S-Trap approach, which showed promising performance in our previous work (25). In light of the significance improvement in phosphoproteome coverage observed with urea supplementation (Fig. 1), we further investigated its effect within SDS-based protocol. Six sample preparation workflows were established for a through comparison (Fig. 2): SDS-based lysis with 5% SDS (SDS) or 5% SDS plus 8 M urea (SU); methanolic lysis with 50% MeOH (ME) or 50% MeOH plus 4 M urea (MUPE, MU), an 8 M urea-only lysis protocol (UA); an S-Trap workflow using 5% SDS with 8 M urea followed by S-Trap micro spin column cleanup (ST). All workflows, except for S-Trap, utilizing MeOH/CHCl_3_ precipitation for protein isolation and contaminant removal. Equal weights of bacterial pellet were processed through each workflow, followed by enzymatic digestion, Fe^3+^-IMAC phosphopeptide enrichment, and LC-MS/MS analysis, as depicted in Figure 2.

Given the consistent phosphopeptide purification procedures across all methods, we hypothesize that the superior phosphoproteomic performance of MUPE approach results from its enhanced protein recovery and more efficient impurity removal. To test this hypothesis, a parallel quantitative proteome analysis was performed on six methods in *L. monocytogenes* prior to phosphoproteome analysis. MeOH-based methods (ME and MU) yielded nearly double the protein amounts compared to the conventional SDS workflow, with MU demonstrating particularly robust performance (Supplemental Fig. S2*A*, and Supplemental Table S1). All workflows identified ∼2000 protein groups and ∼20,000 peptide groups, with comparable protein/peptide identification, sequence coverage, and abundance distributions for shared proteins (Supplemental Fig. S1, *B*–*E* and Supplemental Table S3), except for SU, which exhibited lower performance. Remarkably, MU demonstrated excellent quantitative reproducibility, with >80% of proteins having CVs <10%, comparable to ST and UA, and superior to SDS and SU (Supplemental Fig. S2*F*). Additionally, over 80% of proteins were consistently identified across all workflows (Supplemental Fig. S2*G*), and the physicochemical properties (theoretical pI, GRAVY index, Supplemental Fig. S2*H*) and functional pathway enrichment (Supplemental Fig. S2*I* and Supplemental Table S3*G*) were similar, confirming that MUPE provides unbiased protein extraction across subcellular structures and protein characteristics.

**Fig. 3:**
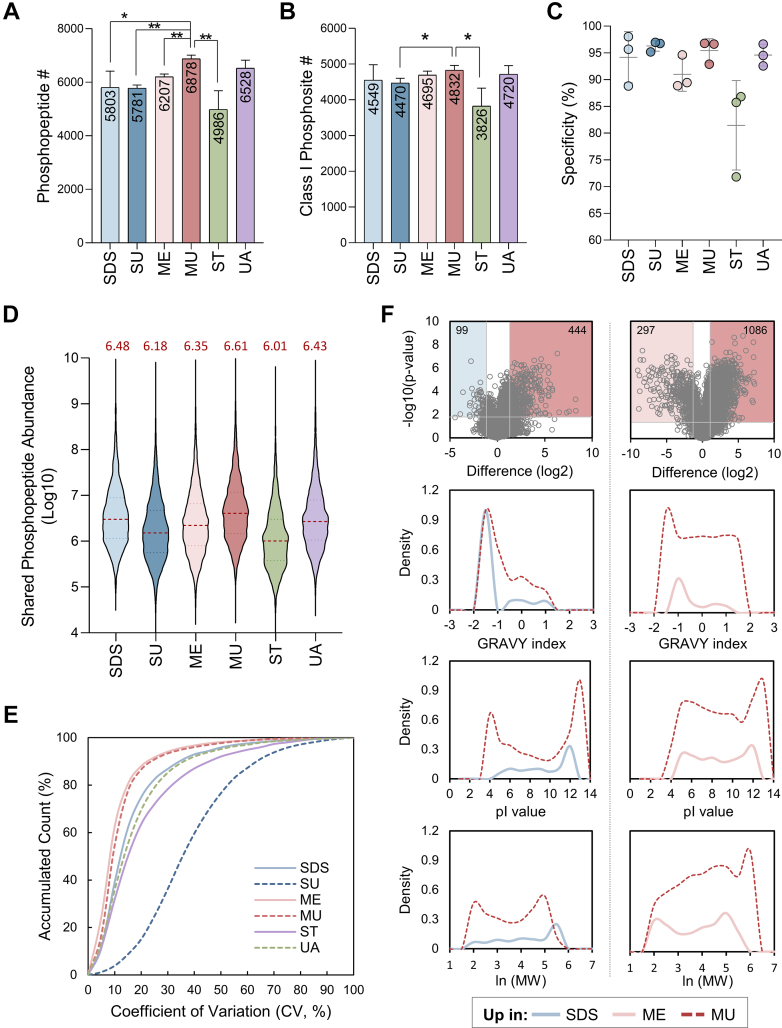
**Assessment of phosphoproteome performance and physicochemical properties of six different protein extraction strategies in *L. monocytogenes*.** Bar chart presented the identification number of (*A*) unique phosphopeptide and (*B*) class I phosphosite across different protein extraction and purification methods. *C*, dot plot showed the specificity (%) of phosphopeptide enrichment. *D*, violin plot depicted the log10-transformed abundances of commonly identified phosphopeptides in technical triplicates across all sample groups (3 valid values). Median intensity was indicated by horizontal *red* dashed line with the value marked on the *top* of each violin. *E*, cumulative distribution of coefficient of variation (CV, %) for quantified phosphopeptides in different protein extraction strategies. *F*, volcano plots showed the comparison of phosphopeptide abundance observed in SDS (*left*) or ME (*right*) compared to MU method. Significant threshold was set as fold change (FC) ≥ 2 and *p*-value <0.05 (*t* test). The *bottom curves* represented the GRAVY index, isoelectric point (pI) and molecular weight with natural logarithm transformation (ln(MW)) of the phosphoproteins bearing statistically differential phosphosites from volcano plots (*up panel*). Y-axis is the density, calculated by dividing the number of differentially phosphorylated proteins (marked on volcano plot) by the number of original identified proteins in each method. All data are presented with mean ± SD and significance was calculated using two sample *t* test (∗, *p* < 0.05; ∗∗, *p* < 0.01). MU was used as our optimized extraction method for data comparison.

Supported by these results, we proceeded to phosphoproteomic analysis to explore phosphorylation dynamics. Notably, the MU workflow outperformed other methods, identifying 7967 phosphopeptides and 5514 class I phosphosites from 1379 phosphoproteins in *L. monocytogenes* (Fig. 3, *A* and *B*, Supplemental Table S4, *A–F*). Notably, MU identified the highest number of unique phosphosites (554), followed by ME (373), reinforcing the enhanced extraction capability of MeOH-based protocols (Supplemental Fig. S3*A*). All workflows, except ST, achieved phosphopeptide enrichment efficiencies above 90%, while ST exhibited greater variability (Fig. 3*C*). To assess the compatibility of the modified S-Trap protocol with urea-containing lysis buffer for bacterial samples, we evaluated two S-Trap workflows: the standard protocol using 5% SDS as the lysis buffer (Strap), and a modified version incorporating 8 M urea (Strap + urea) (25), as illustrated in Supplemental Fig. S4*A*. Strap + urea showed comparable phosphopeptide identification and improved quantitation relative to Strap, however, both S-Trap methods underperformed compared to the conventional SDS workflows (Supplemental Fig. S4*B* and Supplemental Table S4, *A*, *E* and *G*). These findings suggest that the suboptimal performance of ST is likely due to the complexity of bacterial samples rather than high urea concentrations. Phosphoacceptor distributions were consistent across all protocols, with pSer, pThr, and pTyr representing approximately 62 to 64%, 27 to 29%, and 8 to 9% of total phosphosites, respectively (Supplemental Fig. S5*A*: pie chart). The ME, MU, and UA workflows exhibited slightly higher phosphosites density per protein and increased phosphorylation multiplicity per phosphopeptides, although these differences were not statistically significant (Supplemental Fig. S5*A*), indicating efficient and unbiased phosphoprotein recovery. Additionally, physicochemical properties of phosphopeptides, including length (8–23 amino acids), charge, and mass-to-charge (*m/z*) ratios (500–1110 Da), were comparable across all methods (Supplemental Fig. S5*B*).

Building on the superior phosphoproteome coverage of the MU workflow, we further assessed its quantitative performance. MU workflow consistently achieved higher median abundances for shared phosphopeptides (Fig. 3*D*), greater reproducibility with over 60% overlap of quantified phosphosites across replicates (Supplemental Fig. S3*B*), and coverage of over 70% of phosphosites detected by other methods (Supplemental Fig. S3*C*). In addition, MUPE’s streamlined protocol minimized sample handling, contributing to its high reproducibility (37, 38, 39, 40, 41, 42), with more than 80% of quantified phosphopeptides showing a coefficient of variation below 15% and Pearson’s correlation coefficients reaching 0.99 between replicates (Figs. 3*E* and Supplemental Fig. S6*A*). In contrast, SDS displayed substantial variability both within and between batches, as evidenced by inconsistent protein yield, identification, and quantification across independent duplicates (Figs. 1 and 3). This may result from the intrinsic resistance of *L. monocytogenes* cell envelope to SDS-mediated lysis (43, 44) and frequent sample transfer. In comparison, the MeOH-based methods, particularly MUPE, showed exceptional intra- and inter-batch consistency, underscoring its robustness and suitability for bacterial phosphoproteomics. Together, these findings establish MUPE as a reliable and effective workflow for in-depth, reproducible phosphoproteomic profiling in bacteria.

We next characterized the physicochemical properties of phosphoproteins with differential phosphopeptide abundances between MU and other methods. As anticipated, the majority of phosphopeptides were more abundance in MU, supporting its superior extraction efficiency of a broader range of phosphoproteins with diverse physicochemical characteristics, specifically, increased hydrophobicity, lower molecular weights, and more acidic isoelectric points (Figs. 3*F*, Supplemental Fig. S6*B*, and Supplemental Table S1). Interestingly, although UA and MU identified similar numbers of phosphopeptides and class I phosphosites, MU consistently yielded higher phosphopeptide abundances, likely due to improved extraction and sample purity by MeOH’s enhanced capability to disrupt bacterial cell walls compared to urea alone. (45, 46, 47). Functional enrichment analysis revealed similar GO cellular component and KEGG pathway profiles across all six workflows, with phosphoproteins predominantly localized in the cytoplasm and plasma membrane, and involved in metabolic and biosynthetic processes (Supplemental Fig. S7). To evaluate the biological relevance of the detected phosphoproteins, we examined phosphorylation events in key bacterial signaling systems, such as carbon metabolism, ABC transporters, phosphotransferase system (PTS), two-component system (TCS), quorum sensing, and other regulatory pathways (48, 49, 50, 51, 52). Notably, MU not only identified a greater number of quantifiable phosphosites within these pathways but also detected a higher density of phosphosites per protein (Supplemental Fig. S8), indicating its outperformed coverage of phosphorylation-mediated regulatory networks compared to other methods. Taken together, these findings demonstrate that MUPE significantly enhances protein extraction without introducing bias, improving both proteome and phosphoproteome analyses. Its streamlined, detergent-free design increases identification depth, quantitative reproducibility, providing a robust platform for studying phosphorylation-dependent signaling in Gram-positive bacteria.

### Wide Feasibility of MUPE for Both Gram-Positive and Gram-Negative Bacteria Significantly Extend the Bacterial Phosphoproteome Resource

**Fig. 4:**
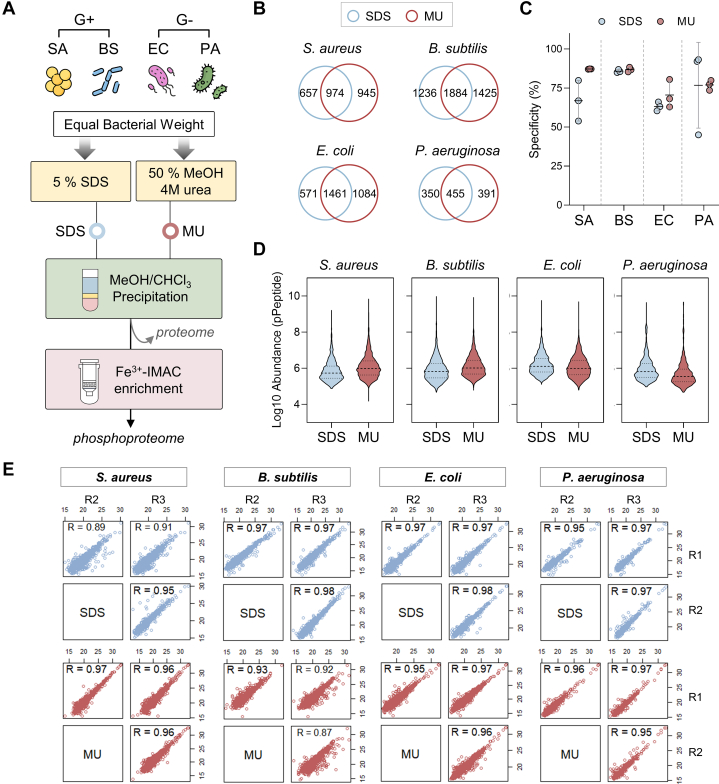
**Evaluation of the performance of MU strategy on both Gram-positive and Gram-negative bacterial phosphoproteomes.***A*, illustration of workflow for comparing the performance between SDS and MU methods on Gram-positive bacteria of *S. aureus* (SA), *B. subtilis* (BS) and Gram-negative bacteria of *E. coli* (EC), *P. aeruginosa* (PA). *B*, Venn diagram showed the overlap number of class I phosphosites between SDS and MU. *C*, dot plot showed the specificity (%) of phosphopeptide enrichment. *D*, distribution of log10-transformed phosphopeptide abundances quantified across all there replicates for each bacterial strain. The *dashed line* in the *middle* of violin indicated the median value. *E*, multiple scatter plots showed the quantitative reproducibility. The Pearson’s correlation coefficient of technical triplicate was labeled on the plot.

To account for the structural diversity of bacterial cell envelopes, which affects lysis efficiency and contaminant profiles (43, 44), we extended the MUPE workflow to four bacterial species: two Gram-positive (*S. aureus*, *B. subtilis*) and two Gram-negative (*E. coli*, *P. aeruginosa*). MUPE was compared to the conventional SDS protocol (Fig. 4*A*). As expected, MUPE achieved proteome coverage comparable to SDS across all species (Supplemental Fig. S9, *A*–*C*). Although SDS lysis yielded slightly higher protein and peptide quantities, MUPE identified more protein groups and achieved greater average protein coverage, suggesting that contaminants in SDS lysis may impair protein and peptide quantification (Supplemental Fig. S9, *A* and *B*, Supplemental Table S1). Venn diagrams showed that MUPE quantified more proteins than SDS lysis, with similar physicochemical properties, pathway enrichment profiles, and reproducibility (Supplemental Fig. S9, *D*–*G*, and Supplemental Table S5). Notably, MUPE consistently outperformed SDS in phosphoproteome coverage across all species (Fig. 4*B* and Supplemental Table S6), exhibiting higher enrichment efficiency (Fig. 4*C*) and a lower missed-cleavage rate (Supplemental Fig. S10*A*), Consistent with prior studies (53, 54, 55), pSer dominated phosphosite distribution, followed by pThr and pTyr, with no significant differences in phosphopeptide multiplicity, protein pI or hydropathy between methods (Supplemental Fig. S10, *B*–*D*). Other than enhanced phosphoproteome coverage, MUPE demonstrated a broader dynamic range of shared phosphopeptide abundance (Fig. 4*D*) and strong reproducibility, with Pearson’s correlation coefficients between replicates from 0.89 to 0.98 (Fig. 4*E*). Collectively, the MUPE workflow enables efficient and reproducible phosphoproteomic profiling across Gram-positive and Gram-negative bacteria, offering a streamlined, detergent-free approach for protein extraction and contaminant removal. Compared to conventional SDS-based methods, MUPE achieves robust cell lysis, higher protein recovery, deeper phosphosite coverage, and enhanced compatibility with Fe^3+^-IMAC phosphopeptide enrichment, resulting in improved phosphosite identification, quantification, and reproducibility. Its simplicity, speed, and versatility position MUPE as a powerful tool for studying phosphorylation-dependent signaling in diverse bacterial species.

### Basophilic and Acidophilic Residues are Extensively Adopted for Mediating Bacterial Phosphorylation Machineries

Using MUPE method, we identified 11,608 Ser/Thr/Tyr phosphosites in *L. monocytogenes*, 2576 in *S. aureus*, 5043 in *B. subtilis*, 3116 in *E. coli* and 1196 in *P. aeruginosa*, constituting the largest bacterial *O*-phosphoproteome dataset reported to date (53, 54, 55, 56, 57) (Supplemental Table S7*A*). Compared to the dbPSP 2.0 (Database of Phosphorylation Sites in Prokaryotes) repository (58), our datasets significantly expand the known bacterial phosphoproteome, covering approximately 35% to 43% of recorded *O*-phosphosites for *B. subtilis* and *E. coli*, respectively (Supplemental Fig. S11*A*). Identified phosphoproteins were primarily enriched in carbohydrate and amino acid metabolism pathways (Supplemental Fig. S11*B*), consistent with prior studies (9, 59, 60). However, species-specific variation in function and pathway enrichment were observed, like due to difference in protein identification depth and the microbial database completeness. The MUPE workflow enabled comprehensive analysis of phosphorylation motif potentially involved in kinase or phosphatase recognition, revealing conserved and species-specific phosphorylation patterns in bacteria, a long-standing gap in prokaryotic signaling research. Due to the limited number of identified pThr and pTyr sites and stringent enrichment criteria, only pSer-containing motifs were significantly enriched via pLogo analysis (34) (Supplemental Fig. S11*C* and Supplemental Table S7*B*). Glutamate (E) and lysine (K) residues were frequently enriched around pSer sites across all species, with glutamate appeared at positions +1 to +4, −1, −3, −4, and −7 in most species, except *P. aeruginosa*, and basophilic residues lysine (K) and arginine (R) were enriched at +4 to +7 and −5 to −7. Species-specific motifs included basophilic lysine-rich motifs in *P. aeruginosa* and acidophilic glutamate-rich motifs in *S. aureus*. These differences may stem from the lower number of identified phosphosites in *S. aureus* and *P. aeruginosa*, which may limit motif diversity and enrichment significance. Additionally, A conserved Gx(pS) motif across all species suggests a shared phosphorylation recognition element critical for bacterial signaling (Supplemental Fig. S11*C*), which may be crucial for conserved bacterial signaling mechanisms.

Accumulating studies highlight the prevalence and functional significance of protein phosphorylation in bacteria, regulating key processes such as differentiation, sporulation, and pathogenicity (61, 62). Unlike eukaryotic phosphorylation, which often features canonical proline-directed or basophilic motifs (14), bacterial motifs are less conserved, with rare exceptions like the x(pT)xEx motif in *Streptomyces* (10, 61). Our datasets show bacteria incorporate acidic and basophilic residues, such as lysine and glutamate, with distinct positional preferences compared to eukaryotes (Supplemental Fig. S11*C*, and Supplemental Table S7*B*), where acidic residues typically appear upstream and basic residues downstream (63, 64). In bacterial, lysine and glutamate are distributed across multiple flanking positions, and glycine may be a distinguishing feature, pending validation. The prevalence of lysine and glutamate suggests bacterial kinases and phosphatases have greater substrate flexibility than eukaryotic counterparts, supporting complex signaling with fewer enzymes. However, the lack of a centralized bacterial phosphorylation database hinders mechanistic insights. This study highlights the need to advance bacterial phosphoproteomics, with the MUPE workflow significantly expanding the phosphorylation landscape across diverse bacterial species, providing a robust resource for investigating phosphorylation-dependent regulatory mechanisms.

### Protein Phosphorylation Serves as a Quicker Responder than Protein Expression upon Bile Challenge in *L. monocytogenes*

*L. monocytogenes* is a foodborne pathogen causing listeriosis, especially dangerous for pregnant women, newborns, the elderly, and immunocompromised individuals (65). Bile’s antimicrobial detergent-like activity pose a threat to non-commensal bacteria, making bile tolerance essential for *L. monocytogenes* to colonize the human gastrointestinal tract (66). While several proteomic studies have elucidated the impact of bile on protein expression (67, 68, 69), the upstream phosphorylation-mediated signaling mechanisms governing bile sensing and stress response remain underexplored. Leveraging the exceptional identification depth and quantitative accuracy of the MUPE workflow (Figs. 3 and 4), we investigated these regulatory mechanisms in *L. monocytogenes* under bile insult with (phospho)proteome analysis (Supplemental Fig. S12*A*). The proteome analysis identified 2106 protein groups at 1% FDR for both peptide and protein identification, with over 80% of proteins showing CVs <25% in treated and untreated samples, confirming high reproducibility across triplicates (Supplemental Fig. S12*B* and Supplemental Table S8*A*). The phosphoproteome analysis detected 3305 class I phosphosites from 4800 phosphopeptides, with pSer, pThr, and pTyr comprising 68%, 25%, and 7%, respectively (Supplemental Fig. S12*C* and Supplemental Table S8*B*). Pearson’s correlation coefficient ranged from 0.95 to 0.97, (Supplemental Fig. S12*D*), establishing a reliable foundation for functional interpretation of bile stress signaling.

**Fig. 5:**
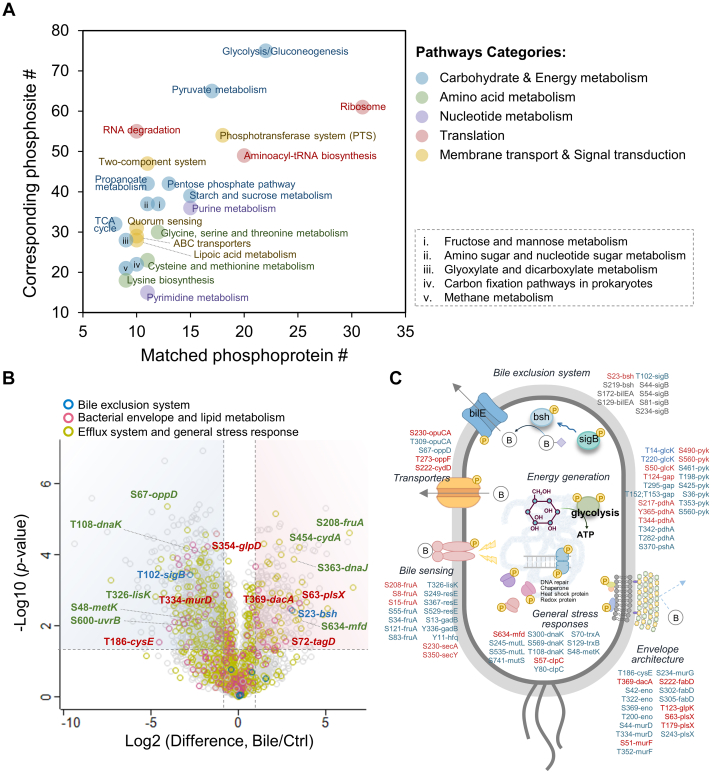
**Phosphoproteome analysis for bile challenge in *L. monocytogenes*.***A*, functional enrichment was performed by using KEGG BlastKOALA for significant regulated phosphoproteins (507 phosphoproteins). X-axis was the number of mapped phosphoproteins, while their corresponding number of phosphosites was indicated in y-axis. *B*, volcano plot of differentially phosphorylated peptides. 279 phosphopeptides were up-regulated in bile treatment and 774 were down-regulated. Student’s *t* test statistic and abundant difference were performed (FDR <0.05, fold change of Bile/Ctrl ≥2). Phosphoproteins involved in bile resistance mechanisms were marked with different colored-circles. *C*, brief scheme of protein phosphorylation in bile resistance and adaptive mechanisms in *L. monocytogenes*. The phosphorylation of key example proteins in each mechanism was listed and highlighted in *red* and *blue* to indicate up- and down-regulation under bile exposure, respectively. Unchanged features were also listed and colored in *gray*.

Quantitative proteomic analysis of *L. monocytogenes* after 30 min of bile exposure revealed minimal changes in protein expression, with only a few cell surface and transcription-related proteins showing significant alterations (Supplemental Fig. S13*A*). In contrast, phosphoproteome analysis uncovered substantial phosphorylation dynamics, with 1597 phosphopeptides significantly perturbed (451 up-regulated and 1146 downregulated, Supplemental Fig. S13*B*). Principal component analysis demonstrated a clear separation between treated and untreated groups, with PC1 explaining 87.7% of the variance (Supplemental Fig. S13*C*). KEGG BlastKOALA pathway enrichment identified significant phosphorylation shifts across five functional categories, including carbohydrate and energy metabolism, translation, nucleotide and amino acid metabolism, membrane transport, and signal transduction (Fig. 5*A* and Supplemental Fig. S13*D*). Notably, glycolysis/glycogenesis, pyruvate metabolism, and stress response systems, including phosphotransferase system (PTS), ABC transporter, two-component system (TCS), and quorum sensing (QS) (70, 71), were prominently affected (Fig. 5*A*, and Supplemental Table S8*C*). Unlike previous transcriptome and proteome studies focusing on prolonged bile exposure (67, 68, 69, 72, 73, 74), our data capture early phosphorylation events in key regulators. Although expression levels of bile exclusion protein (bilE) (75), stress-response regulator sigma B (sigB) (76, 77, 78), and bile salt hydrolase (bsh) (79, 80, 81) remained unchanged (Supplemental Fig. S13*A*), their phosphorylation states were significantly altered, with notable modulation at Ser23 on Bsh (near the catalytic loop) (81) and Thr102 on SigB (within the conserved transcription initiation domain) (78) (Fig. 5*B* and Supplemental Table S8*B*). These site-specific changes suggest rapid post-translational regulation, possibly enabling *L. monocytogenes* to mount an immediate defense against bile stress prior to transcriptional responses.

Beyond the bile exclusion systems, the bacterial envelope serves as a primary barrier against external compounds. Bile exposure is known to alter its architecture by impacting proteins involved in peptidoglycan biosynthesis and fatty acid metabolism (82, 83), thereby enhancing mucus adhesion and limiting bile diffusion to support *L. monocytogenes* intestinal colonization and survival (66, 70). From our analysis, we also identified significant phosphorylation changes in *L. monocytogenes* cell envelope proteins under bile stress, such as Thr369 on dacA and Thr334 on murD (peptidoglycan synthesis) and Ser63 on plsX and Ser354 on glpD (fatty acid metabolism). Additionally, bile exposure modulated the phosphorylation of proteins linked to stress responses, such as efflux systems (ABC transporters, TCS, PTS, and QS), DNA repair, chaperone activity, and oxidative stress responses (Fig. 5*B* and Supplemental Table S8*C*). These findings elucidate phosphorylation-mediated mechanisms of bile sensing and adaptation, offering novel insights for developing targeted therapeutic strategies against *L. monocytogenes*.

## Discussion

Despite ongoing advancements in MS-based phosphoproteomics, the progress of bacterial phosphoproteome analysis still lags and lacks a one-size-fits-all solution. Drawing on organic solvent-based LLE, commonly used to separate lipids, metabolites, and proteins from complex biological samples, we developed the MUPE method. This detergent-free, user-friendly, and rapid approach eliminates the need for sample transfer and complex manipulations, streamlining the process for more efficient analysis. By optimizing the amphiphilic nature of methanol and the chaotropic properties of urea, MUPE enhances protein recovery and interference removal, enabling efficient phosphopeptide purification for system-wide bacterial phosphoproteome studies. Our results demonstrate that MUPE provides superior protein yields and quantitative accuracy in both proteome and phosphoproteome analyses (Figs. 3 and 4, Supplemental Fig. S2 and S9, and Supplemental Table S1), significantly outperforming SDS-based methods in expanding phosphoproteome coverage across both Gram-positive and Gram-negative bacteria. Of noted, MUPE shows consistent performance across intra- and inter-batch comparisons (Figs. 3 and 4), making it particularly suitable for sample with limited materials, such as clinical biopsies or dental plaque. Furthermore, its rapid, solvent-based approach holds promise for investigating acid-labile arginine and histidine phosphorylation, which play key roles in prokaryotic signal transduction (15, 84, 85, 86). Thus, MUPE represents a robust platform for advancing bacterial phosphoproteomic research.

With this efficient MUPE approach, we generated a comprehensive bacterial *O*-phosphorylation resource (∼21,000 sites), revealing a pronounced preference for acidic and basic residues flanking phosphoserine sites across multiple bacterial species (Supplemental Fig. S11*C* and Supplemental Table S7*B*). While such motifs are well-documented in eukaryotic cells, their distinct positioning in bacteria suggest potential targets for antibiotics development. Comparing to recent phosphoproteomic study in *Mycobacterium tuberculosis* (87), MUPE achieved comparable phosphosite coverage with reduced sample input and shorter MS analysis time (Supplemental Table S7*A*), reinforcing its efficiency. Although our dataset overlaps only 35 to 50% of entries in dbPSP 2.0 (Supplemental Fig. S11*A*), this likely reflects variations in culture conditions, stimuli, and enrichment preferences. Collectively, these findings demonstrate MUPE’s capability to significantly expand the bacterial phosphoproteome landscape, advancing our understanding of phosphorylation-driven regulations in prokaryotes and offering valuable insights for novel antimicrobial strategies.

While it is hypothesized that bacterial stress signals are initiated by perturbations in *O*-phosphorylation, followed by changes in protein expression, direct evidence has been limited. Utilizing MUPE approach, we demonstrated that *L. monocytogenes* stress responses to bile salt exposure mirror eukaryotic systems, with *O*-phosphorylation enabling rapid adaptive responses (Fig. 5). This represents the first well-documented case of such a mechanism in bacteria, made possible by MUPE’s depth and quantitative accuracy in bacterial phosphoproteomics, overcoming previous technical limitations. MUPE offers a rapid, highly reproducible, and broadly applicable platform for decoding phosphorylation-mediated signaling in bacteria, illuminating a largely unexplored frontier in phosphoproteomics. Notably, while MeOH/CHCl_3_ and MeOH/MTBE (methyl-tert-butyl-ether) extractions are widely employed in metabolomics and lipidomics (88, 89, 90), their application in proteomics remains underexplored. As the focus shifts toward integrated multi-omics, methods such as MPLEx (91), SIMPLEX (92), and SPM-LLE (93) have emerged to co-extract metabolites and proteins from a single sample using similar solvent systems. While MUPE has proven highly effective for bacterial phosphoproteomics, our study focused solely on MeOH as the detergent alternative. Further optimization, including exploring alternative solvents or combined extraction strategies, will be crucial to enhance its versatility and applicability in (phospho)proteomics and multi-omics workflows.

## Data Availability

All MS raw data and search result files that support the findings of this study have been deposited in jPOST repository (94) (https://repository.jpostdb.org). The accession numbers are PXD055159 for ProteomeXchange and JPST003043 for jPOST.

## Supplemental Data

This article contains supplemental data (53, 54, 55, 56, 57, 87).

## Conflict of interest

The authors declare that they have no conflicts of interest with the contents of this article.
