# Supplementary material for: A Methanolic Urea-Enhanced Protein Extraction Enabling the Largest Bacterial Phosphorylation Resource
Source: Mol Cell Proteomics. 2025 Jun 24;24(8):101019. doi: 10.1016/j.mcpro.2025.101019 (PMC12345307; doi:10.1016/j.mcpro.2025.101019)
Supplement: Supplemental Information [file mmc1.pdf]

Supplemental Information

A Methanolic Urea-enhanced Protein Extraction Enabling the Largest Bacterial Phosphorylation Resource

Pei-Shan Wu [1,2], Ting-An Chen [1], Bo-Yu Chen [1], Yasushi Ishihama [2,3], Miao-Hsia Lin [1*]

Supplemental information file content

1. Figure S1
2. Figure S2
3. Figure S3
4. Figure S4
5. Figure S5
6. Figure S6
7. Figure S7
8. Figure S8
9. Figure S9
10. Figure S10
11. Figure S11
12. Figure S12
13. Figure S13
14. Experimental Protocol: SDS-based protein extraction (SDS and SU)
15. Experimental Protocol: MeOH-based protein extraction (ME and MU)
16. Supplementary Tables (.xlsx)

    Table S1.
    Table S2. A-H related to Fig. 1
    Table S3. A-G related to Fig. S2
    Table S4. A-G related to Fig. 3
    Table S5. A-I related to Fig. S9
    Table S6. A-H related to Fig. 4
    Table S7. A-B related to Fig. S11
    Table S8. A-C related to Fig. 5

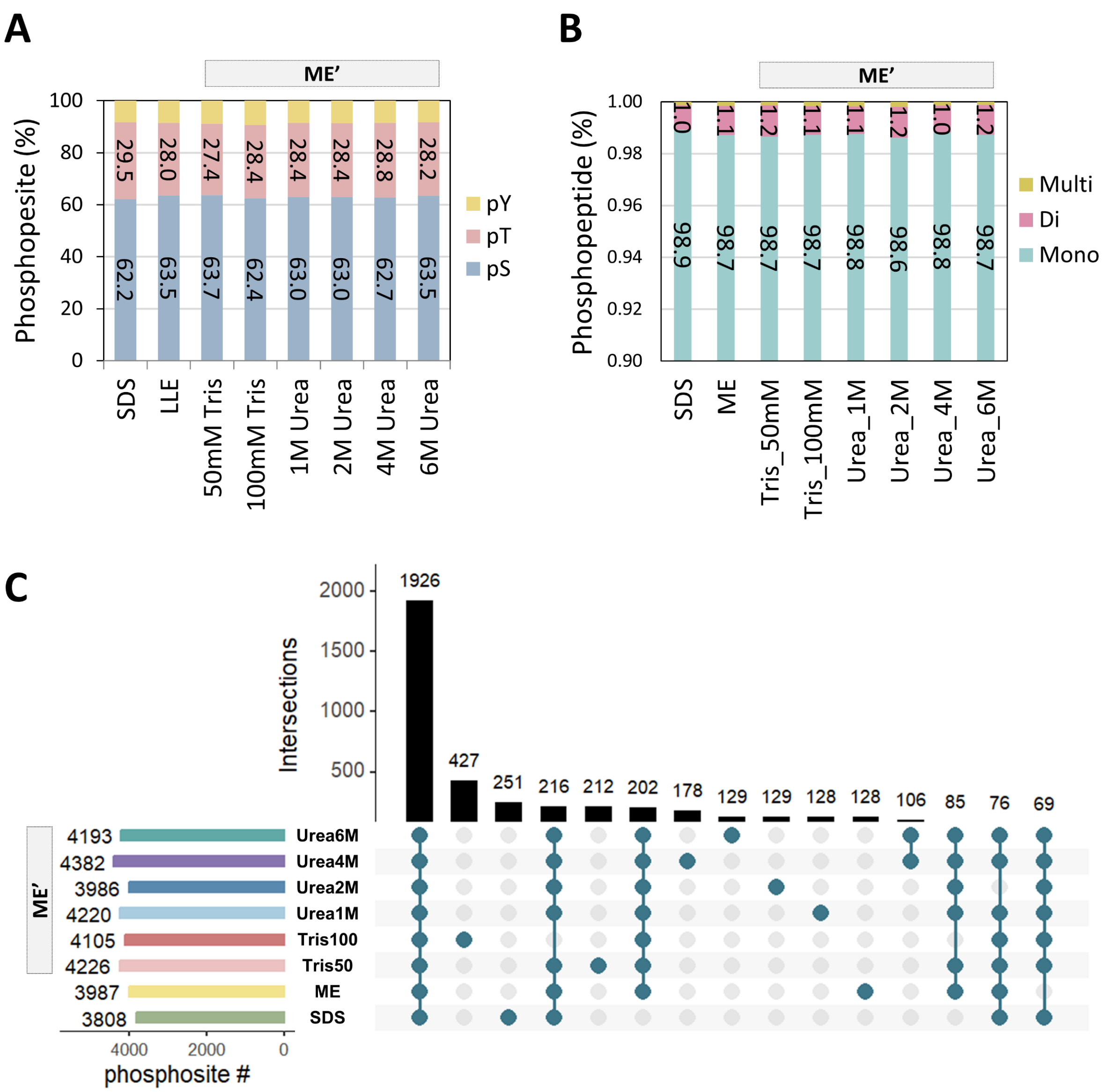

Fig. S1. Evaluation the phosphoproteome performance of ME and modified ME' workflows in *L. monocytogenes*. Cumulative bar charts depicted the percentage of the distribution of (A) class I phosphosites (localization probability > 0.75) on serine (pS), threonine (pT), and tyrosine (pY) residues (top-panel), and (B) the composition of singly (Mono), doubly (Di) and multiply (Multi) phosphorylated peptides (bottom-panel). (C) The upset plot showed the intersection of class I phosphosites identified across all six methods. The horizontal bar graphs showed the number of phosphosites identified in at least two times from triplicate experiments. The vertical bar graphs illustrated the number of commonly identified phosphosites in the experiments indicated in the bottom joined circles. SDS, the conventional SDS-based lysis workflow; ME, the methanol-alone cell lysis workflow; ME', the workflow with the addition of different concentration of Tris or urea to methanol for cell lysis (50 / 100 mM Tris and 1 / 2 / 4 / 6 M Urea).

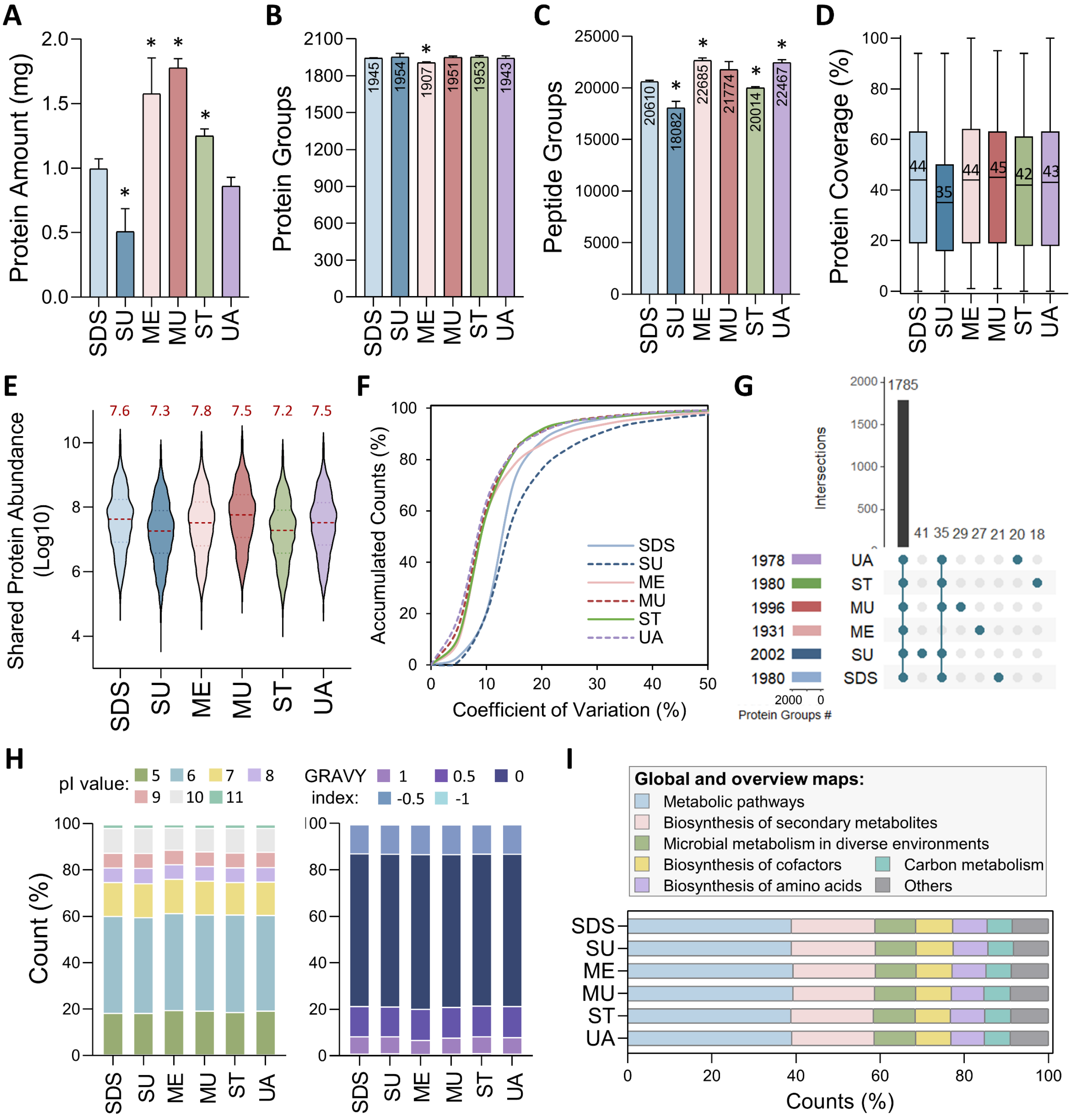

Fig. S2. Characterization of proteome for six protein extraction strategies in *L. monocytogenes*. (A) Bar charts showing protein yields from equal bacterial pellet weights. (B, C) Bar charts displaying the average numbers of identified protein groups and peptide groups. All data are presented as mean ± SD (N=3), with statistical significance determined using a two-sample t-test (*p-value < 0.05). (D) Box plot illustrating the percentage of sequence coverage for identified proteins, with the median value indicated at the center of the box. (E) Violin plot depicting the distribution of log10-transformed abundance of commonly identified proteins across all sample groups in technical triplicate (3 valid values). The red dashed line represents the median value. (F) Cumulative distribution of coefficient of variation (CV, %) for quantified proteins across six sample groups. (G) Upset plot showing the overlap of identified proteins across six extraction methods. (H) Stacked bar charts illustrating the distribution of theoretical pI values and GRAVY indices of identified proteins. (I) Pathway enrichment analysis of identified proteins using KEGG KOALA.

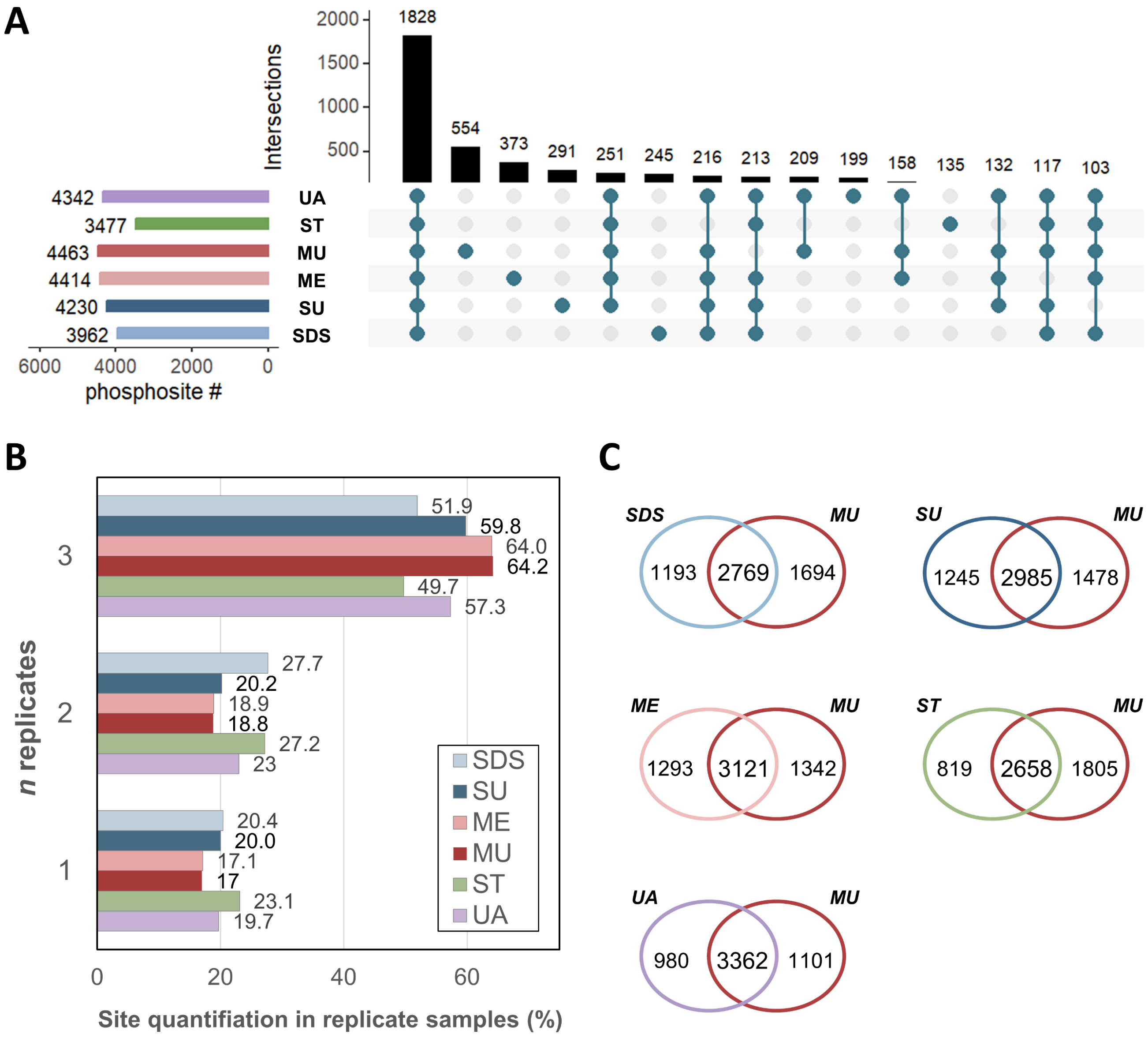

Fig. S3. Overlap of quantified phosphosites among protein extraction methods in *L. monocytogenes*. (A) Upset plot depicting the intersection of phosphosites across six methods. Horizontal bar graphs show the number of quantifiable phosphosites (with 3 valid values) for each method. Vertical bar graphs illustrate the number of commonly identified phosphosites in the sample groups indicated by the joined circles at the bottom. (B) Distribution of quantified phosphosites in *n* replicate samples. (C) Venn diagram illustrating the number of quantifiable phosphosites identified in MU and other methods (3 valid values).

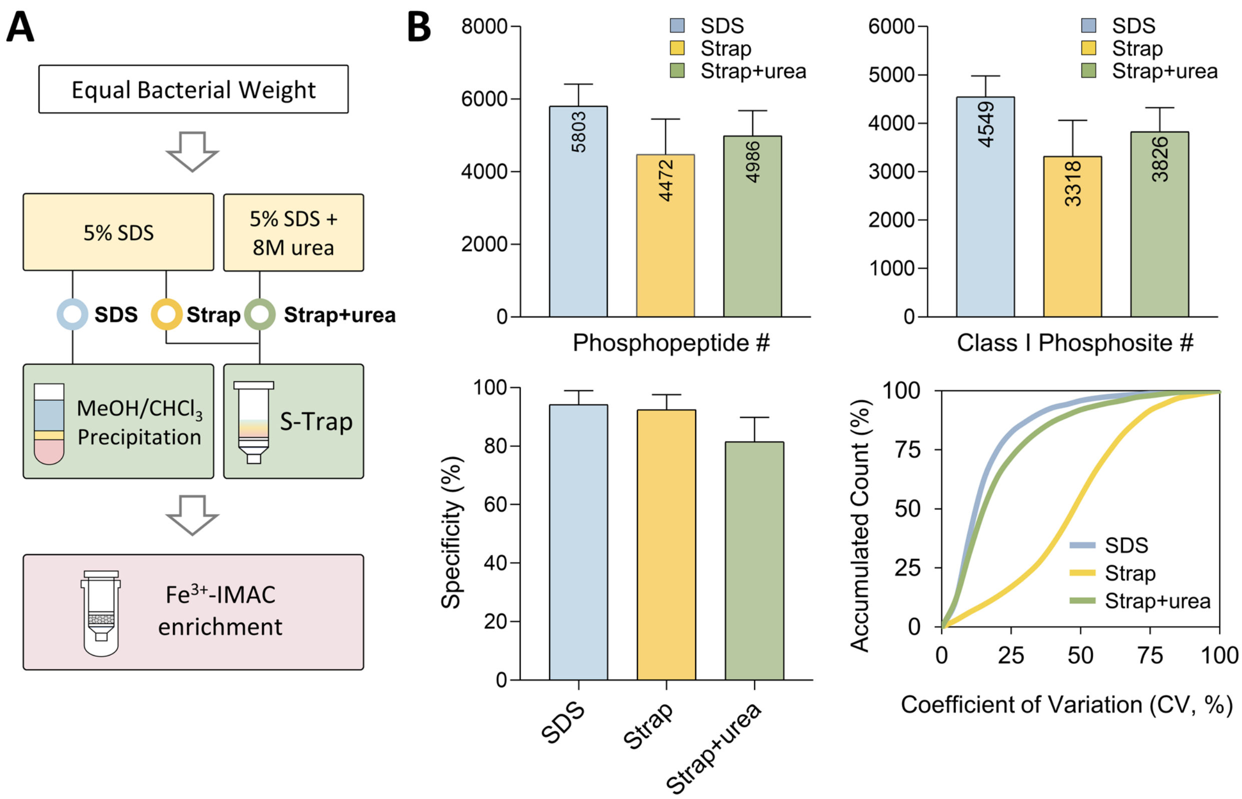

Fig. S4. Comparison of phosphoproteome performance between SDS and S-Trap in *L. monocytogenes*. (A) Schematic illustrating the workflows for SDS and S-Trap protocols, using lysis buffer with (S-Trap + urea) or without (S-Trap) 8 M urea. (B) Bar charts represented the number of identified phosphopeptides and class I phosphosites. Phosphopeptide enrichment specificity (%) is presented as a dot plot, and the coefficient of variation (CV, %) is depicted as a cumulative distribution curve. All data are presented as mean ± SD (N=3).

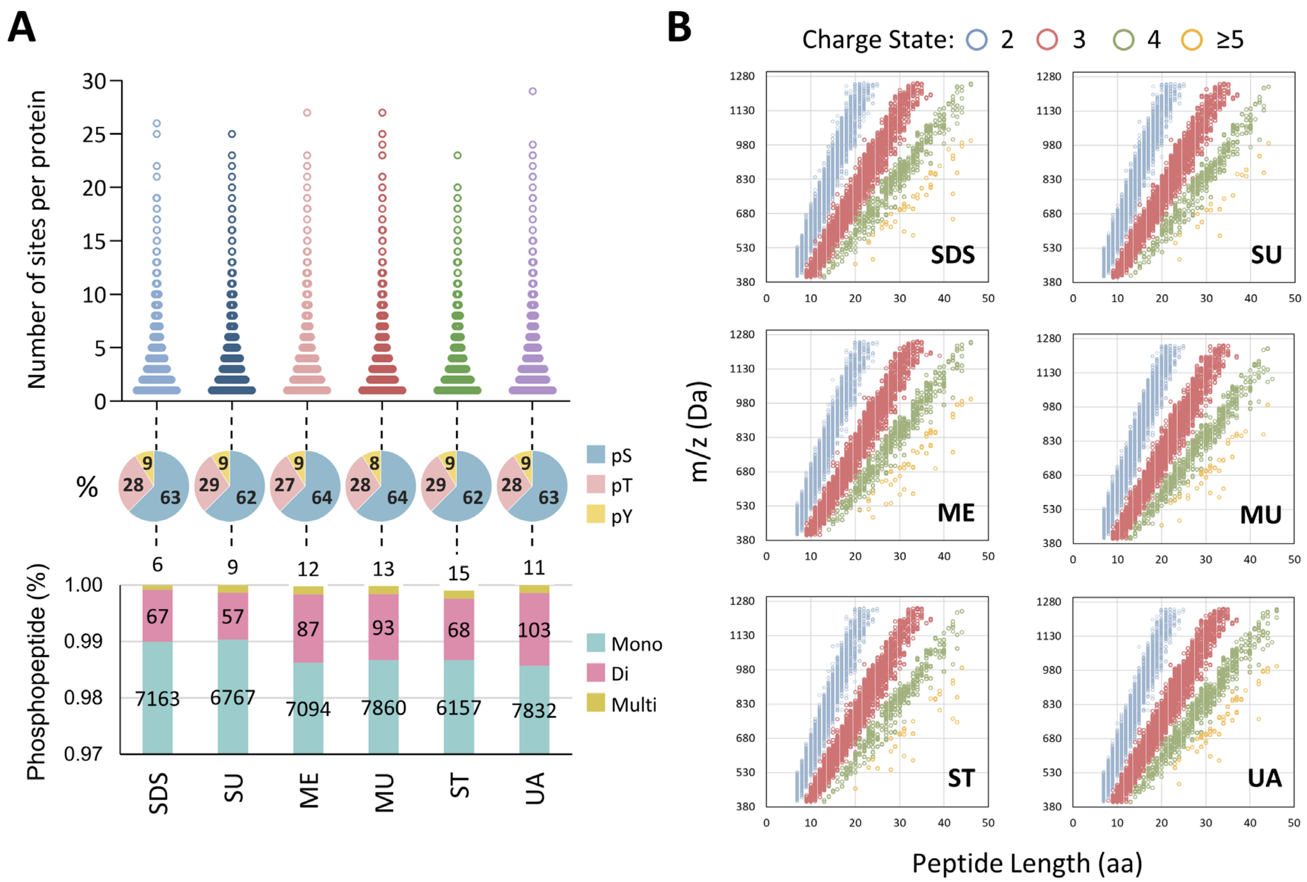

Fig. S5. Characterization of phosphoproteome features using six different protein extraction approaches in *L. monocytogenes*. (A) Dot plot represented the distribution of phosphorylation sites per protein (top panel) and pie chart meant the percentage composition of serine (pS), threonine (pT) and tyrosine (pY) phosphorylation residues from the identified class I phosphosites (middle panel). Cumulative bar indicated the distribution of of singly (Mono), doubly (Di) and multiply (Multi) phosphorylated peptides (bottom panel). (B) Distribution of identified phosphopeptides derived from six protein extraction methods, plotted as peptide length versus m/z and color-coded by charge state.

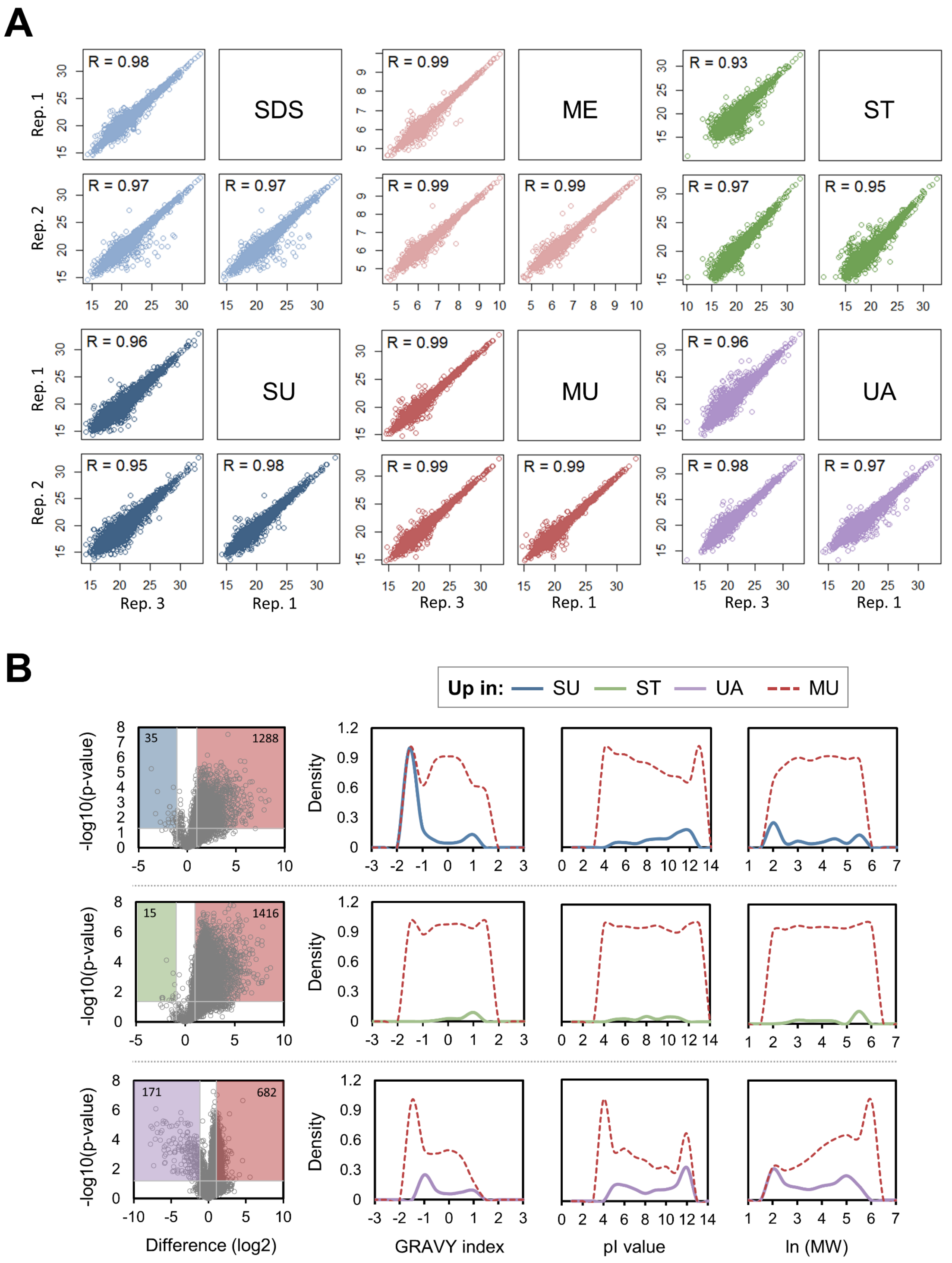

Fig. S6. Comparison of quantitative reproducibility and physiochemical features of phosphoproteins with differentially abundant phosphosites using six protein extraction strategies in *L. monocytogenes*. (A) Multiple scatter plots showed the correlation of quantified phosphopeptides between technical triplicate and Pearson's correlation coefficient was labeled on the plot. (B) Volcano plots mean the comparison of phosphopeptide abundance observed in SU (up), ST (middle), and UA (bottom) compared to our optimized MU workflow. Significant threshold was set as fold change (FC) ≥ 2 and *p*-value < 0.05 (*t*-test). The right curves showed the GRAVY index, isoelectric point (pI) and molecular weight with natural logarithm transformation (ln(MW)) of the phosphoproteins bearing statistically differential phosphosites from volcano plots. Y-axis is the density, calculated by dividing the number of differentially phosphorylated proteins (marked on volcano plot) by the number of original identified proteins in each method.

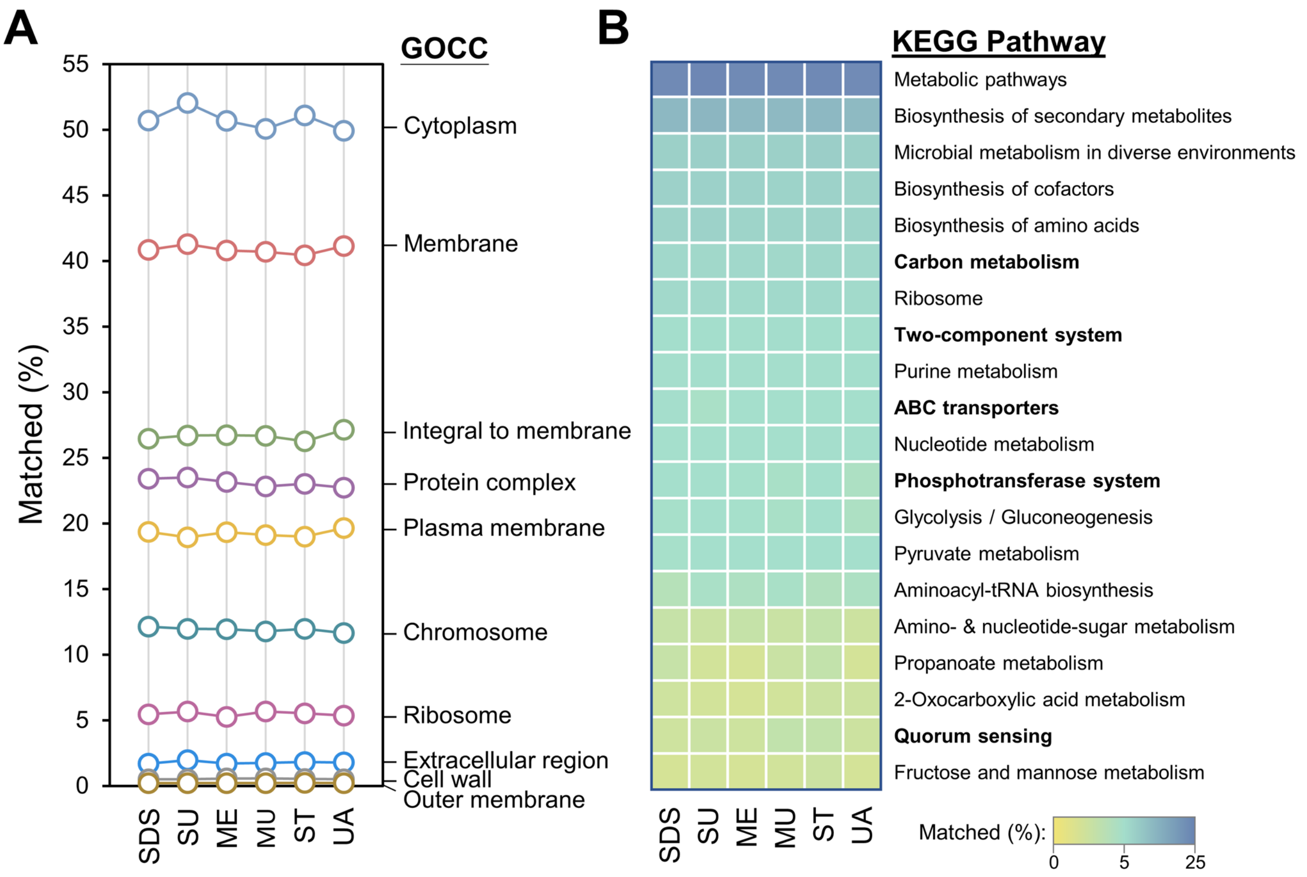

Fig. S7. Functional annotation and pathway enrichment of phosphoproteins identified by six different protein extraction methods in *L. monocytogenes*. (A) Gene Ontology annotation analysis of cellular component (GOCC). (B) Top 25 KEGG pathways mapped by KEGG BlastKOALA. Matched percentage (%) was calculated by dividing the number of mapping entries by the total number of entries in each method.

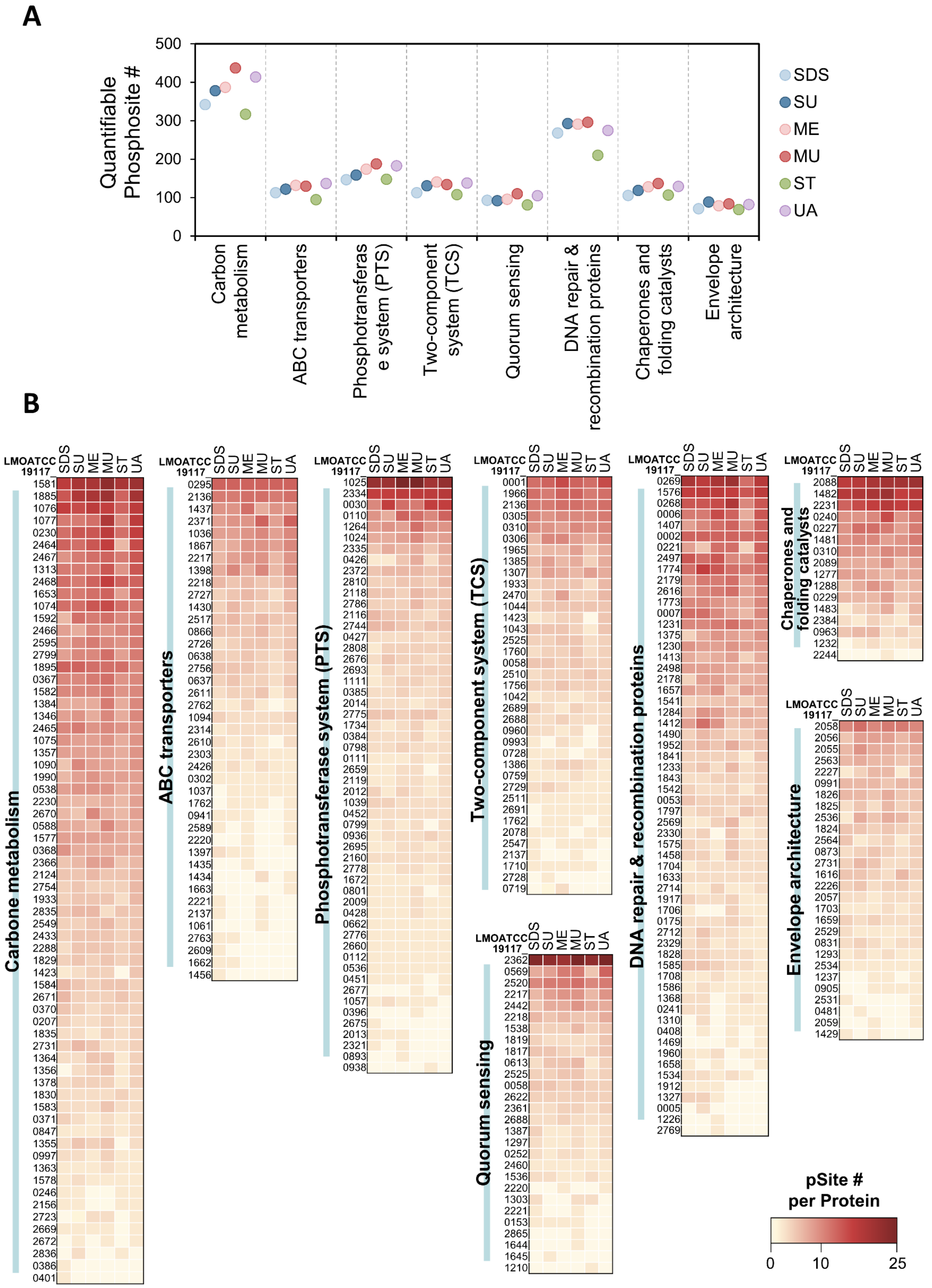

Fig. S8. Pathway mapping for quantifiable phosphosites identified by six protein extraction methods. (A) Dot plot showing the total number of quantifiable phosphosites for corresponding phosphoproteins within specific pathways. (B) Heatmap illustrating the number of quantifiable phosphosites for each phosphoprotein within specific pathways. The x-axis represents experimental sample groups for the six protein extraction methods, and the y-axis lists phosphorylated protein IDs from *L. monocytogenes*. Pathway mapping was performed using BlastKOALA.

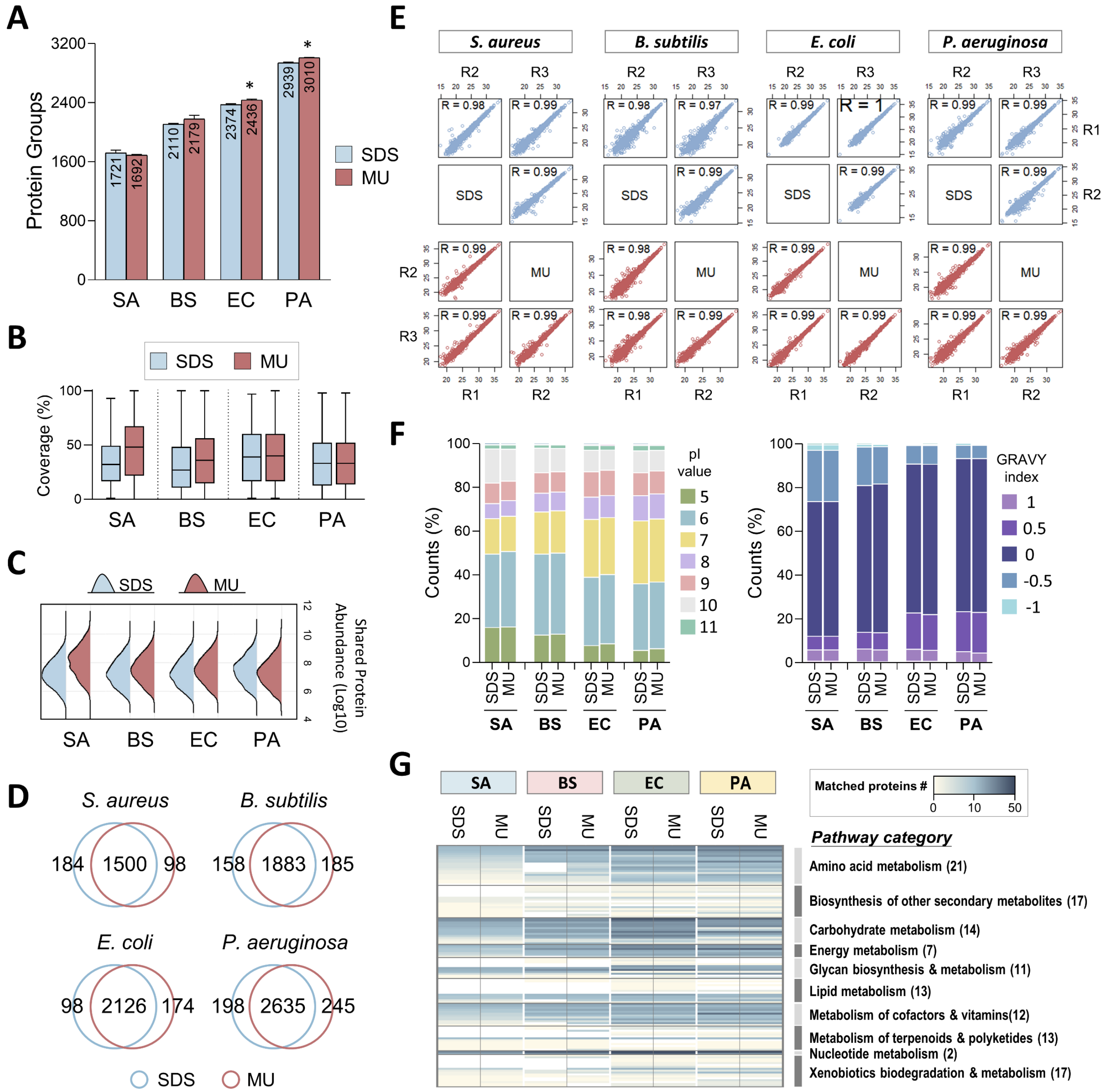

Fig. S9. Characterization of proteins identified using SDS and MU methods in both Gram-positive and Gram-negative bacteria. Proteome analysis were performed on *S. aureus* (SA), *B. subtilis* (BS), *E. coli* (EC), and *P. aeruginosa* (PA). (A) The bar charts illustrated the number of identified protein groups. Data were presented with mean ± SD and significance was calculated using two sample *t*-test (**p*-value < 0.05). (B) Box plot displayed the percentage of sequence coverage for identified proteins. The middle line in the box represented the median value. (C) Distribution of log10-transformed abundance for quantifiable proteins commonly identified between SDS and MU (*N* = 3, all with valid values). (D) Venn diagram showing the overlap number of quantifiable proteins identified in SDS and MU. (E) Multiple scatter plots showed the quantitative reproducibility with Pearson's correlation coefficient of technical triplicate indicated on each plot. (F) Distribution of theoretical pI value and GRAVY index of identified protein groups. (G) Proteins identified from SDS and MU were subjected to pathway mapping using KEGG BlastKOALA. The heatmap illustrated the categorized pathways enriched in each sample group. Numbers in parentheses denote enriched pathway counts.

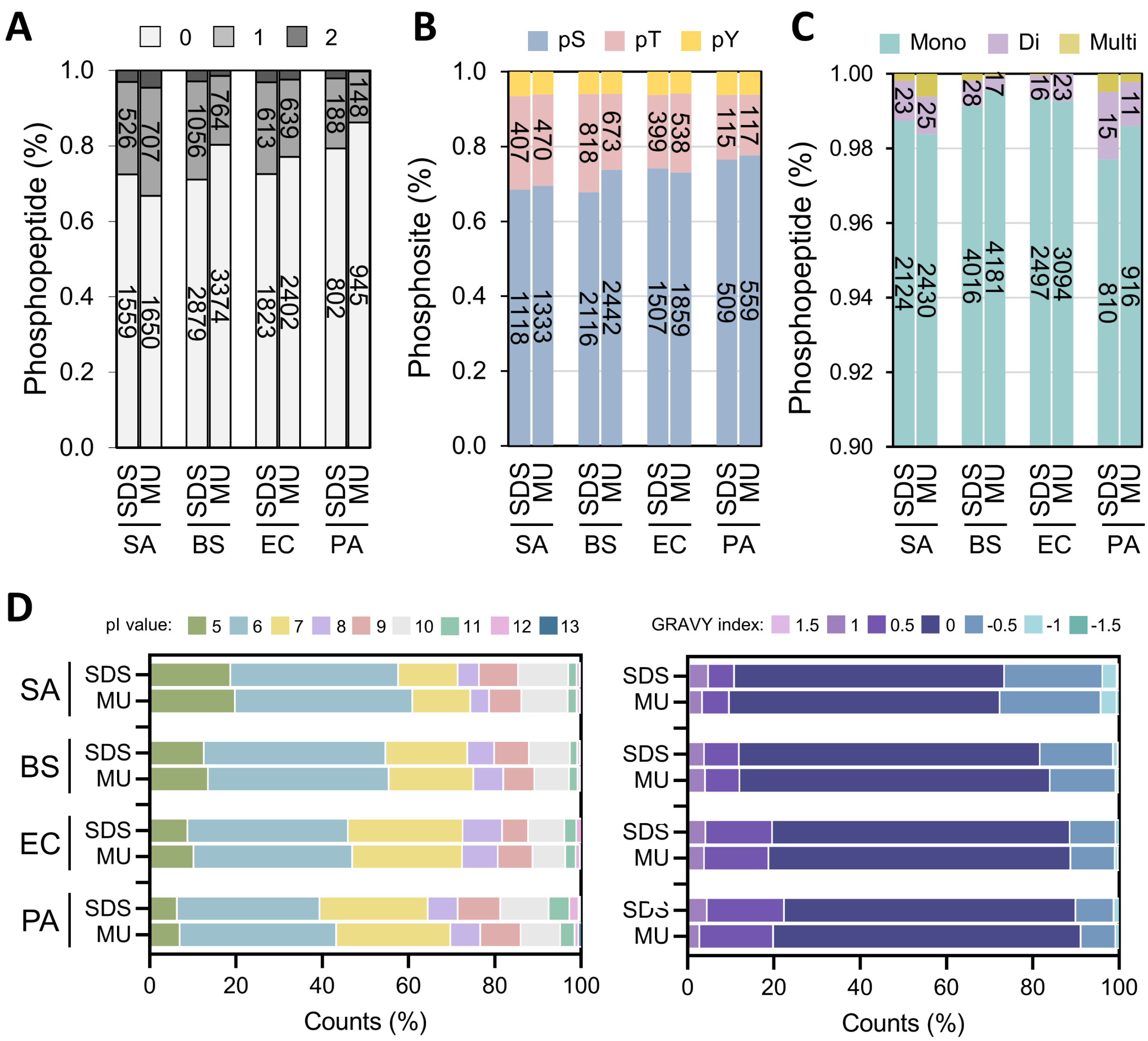

Fig. S10. Characterization of phosphoproteome properties in Gram-positive and Gram-negative bacteria using SDS and MU approaches for protein extraction. The cumulative bar charts showed (A) the distribution of missed-cleavage phosphopeptides, (B) phosphorylated residues at serine (pS), threonine (pT), and tyrosine (pY) (left), (C) singly (Mono), doubly (Di) and multiply (Multi) phosphorylated peptides (right), and (D) theoretical pI value and GRAVY index of identified phosphoproteins. The number of identified phosphopeptides or phosphosites was marked in the center of accumulated bar. SA: *S. aureus*, BS: *B. subtilis*, EC: *E. coli*, PA: *P. aeruginosa*.

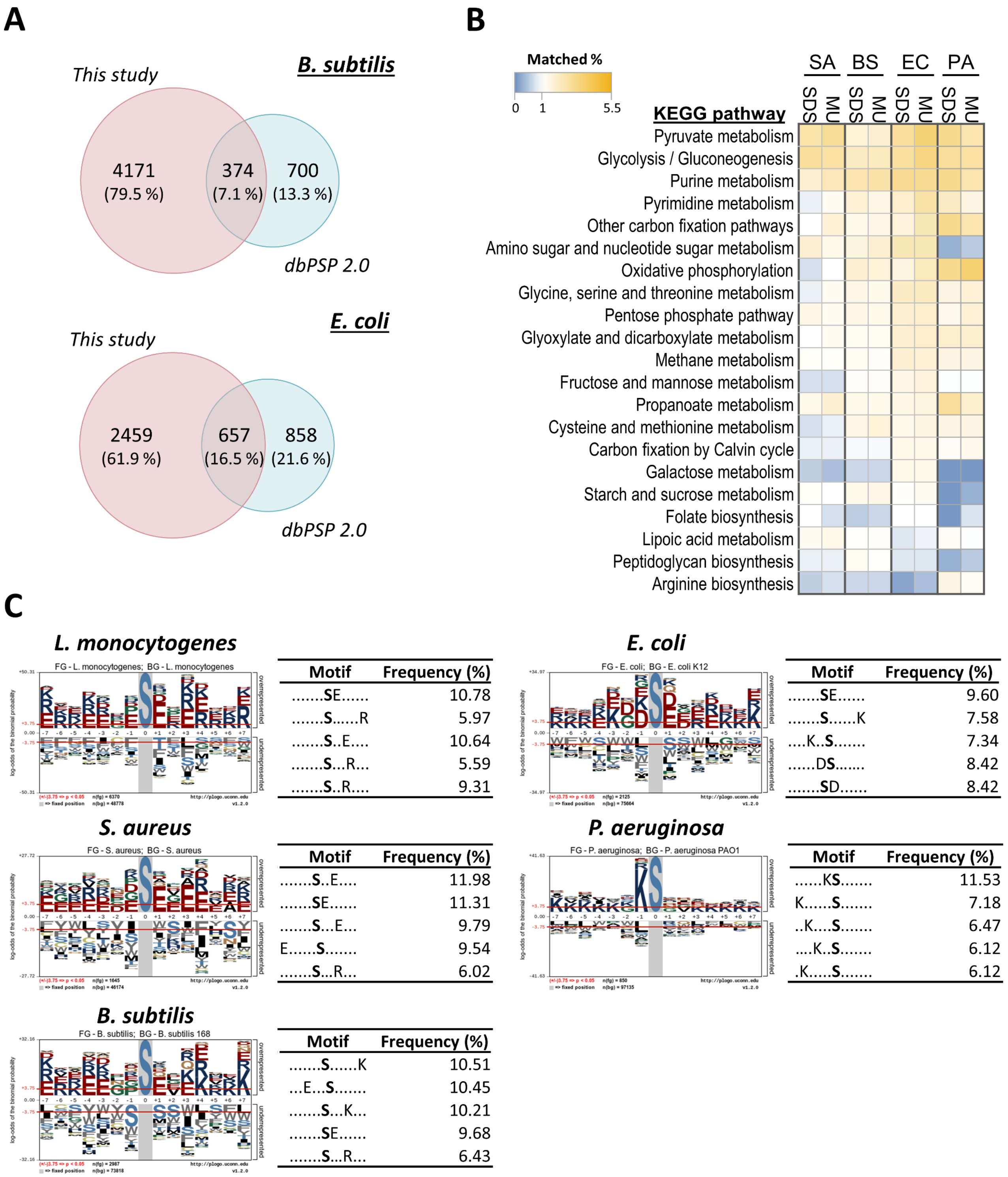

Fig. S11. Characterization of the compiled phosphoproteome atlas observed from five bacterial models. (A) Comparison of Ser/ Thr/ Tyr phosphoproteome coverage of the model organisms *E. coli* and *B. subtilis* in this study with dbPSP 2.0 (database of Phosphorylation Sites in Prokaryotes). (B) Phosphoproteins identified in SDS and MU were subjected to pathway enrichment by using KEGG BlastKOALA. Top20 matched pathways were presented using a heatmap with color barcode indicating the percentage of matched protein numbers out of the total identification. SA: *S. aureus*, BS: *B. subtilis*, EC: *E. coli*, PA: *P. aeruginosa.* (C) Representative motif sequence of total identified phosphoserine (pS) in each bacterial species. Motif logo was generated using pLogo and the table listed the Top5 significant enriched positions and their corresponding frequency (%).

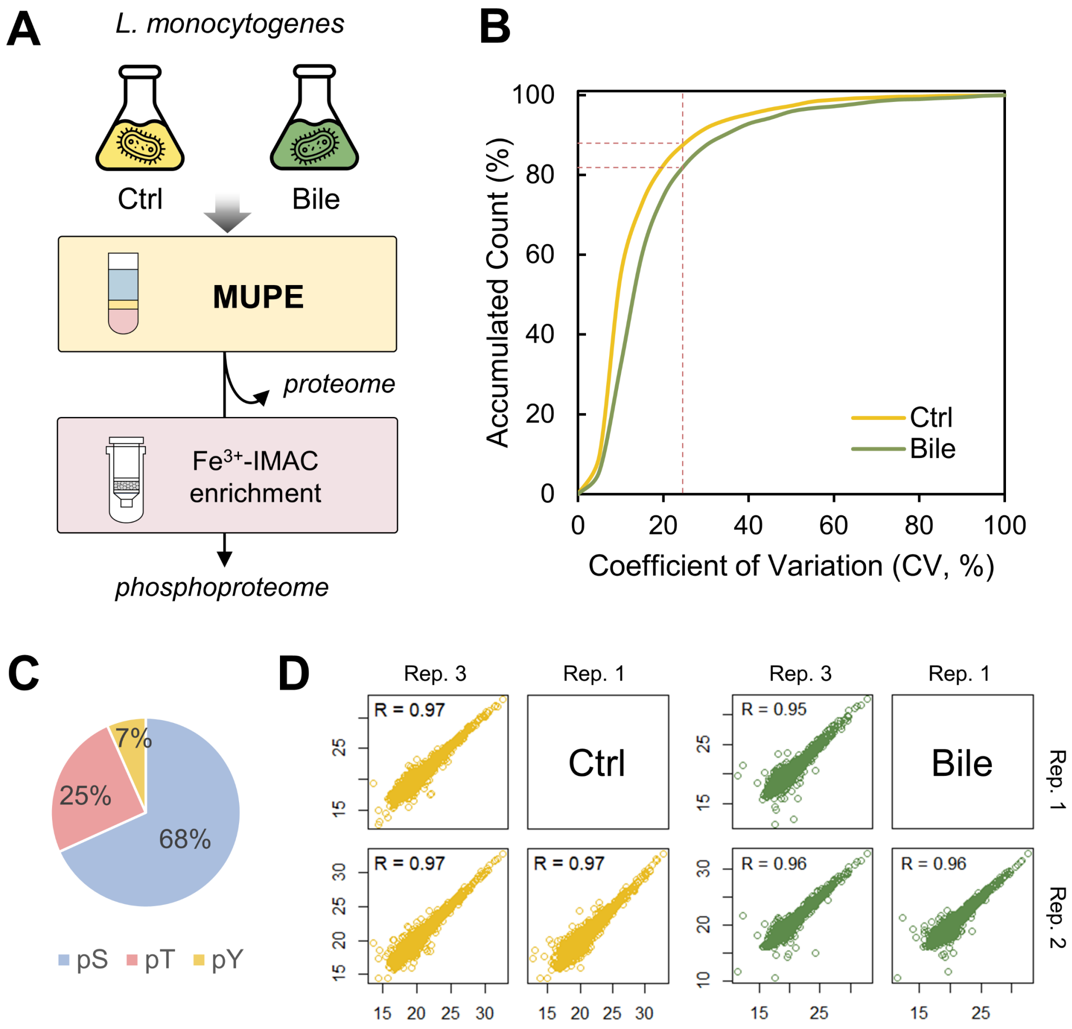

Fig. S12. (Phospho)proteome analysis on bile-treated *L. Monocytogenes.* (A) Workflow of (phospho)proteomics analysis on *L. monocytogenes* treated with or without bile. (B) Cumulative distribution of coefficient of variation (CV, %) for quantified proteins in Ctrl (yellow) and Bile (green) groups across biological triplicates. (C) Pie charts showed the distribution of phosphorylated serine (pS), threonine (pT) and tyrosine (pY) residues of identified class I phosphosites. (D) Multiple scatter plots indicated the correlation between biological triplicate in (yellow) and Bile (green) groups with the Pearson's correlation coefficient was labeled on the plot.

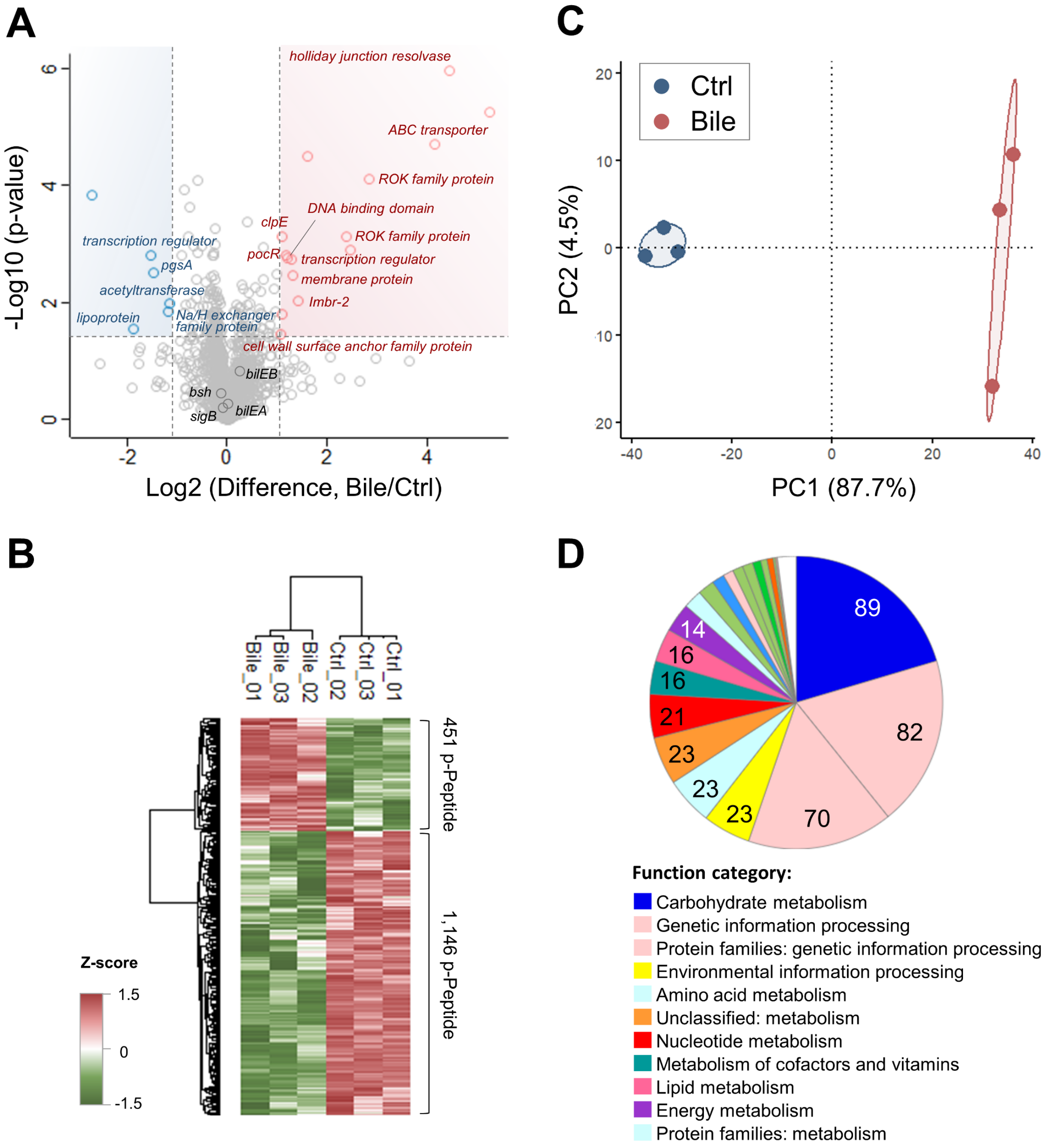

Fig. S13. Label-free quantitative (phospho)proteome comparisons in bile-treated *L. monocytogenes*. (A) Volcano plot showed the differentially expressed proteins between Ctrl and Bile samples. Circles in pink and blue area indicate significantly up-regulated (6 proteins) and down-regulated (15 proteins) expressions in response to bile treatment, respectively. (B) Hierarchical clustering of significantly regulated phosphopeptides (451 up-regulated phosphopeptides, 1,146 down-regulated phosphopeptides). The color code shows the relative abundance based on the Z-score transformed intensity. (C) Principal component analysis of the differentially phosphorylated peptides between Ctrl and Bile groups. (D) The functional categories of 436 out of 507 phosphoproteins carrying the 1,597 differentially phosphorylated peptides were annotated using KEGG BlastKOALA. The numbers of matched phosphoproteins ≥ 10 were indicated on the pie chart.

Experimental Protocol: SDS-based protein extraction (SDS and SU)

1. Protein extraction

(1) An equal weight of bacterial pellet was lysed with 5 % SDS in 50 mM TEABC, either with 8 M urea (SU) or without urea (SDS). The buffer volume used was 40 μL per 10 mg of pellet.

(2) The well-mixed sample was submitted for homogenization using Bioruptor Plus with frequency of 30s on/ 30s off per cycle, high energy mode, under 4 ℃. A total of 40 cycles were applied for Gram-positive bacteria, while 25 cycles were used for Gram-negative bacteria. (note: Only the SDS sample need to be heated at 95 ℃ for 5 mins prior to homogenization.)

(3) To remove cell debris, homogenized sample was centrifuged at 17,000 x g for 30 mins under 4 ℃.

(4) The supernatant was collected and subjected to MeOH/$CHCl_3$ protein precipitation. The supernatant was added with 4-fold its volume of MeOH, 1-fold its volume of $CHCl_3$, and 3-fold its volume of H2O, followed by vertexing to ensure thorough mixing. (note: final ratio of MeOH : $CHCl_3$: H_2O = 4 : 1 : 3, v/v/v)

(5) The protein pellet was collected by centrifugation at 17,000 xg for 10 mins at RT and the white protein precipitate was allowed to air dry and dissolved with 8 M urea in 50 mM TEABC.

(6) The protein concentration was determined using BCA assay, the following manufacturer's guidelines.

2. In-solution protein digestion and peptide desalting

(1) Protein extract in 8 M urea/50 mM TEABC was reduced and alkylated at 29 ℃ by 10 mM TCEP for 30 mins and 40 mM CAA for 45 mins, respectively.

(2) The sample was diluted with 50 mM TEABC to a urea concentration of 4 M, followed by digestion with Lys-C (1:100, w/w) for 3 hrs at 29 ℃.

(3) The sample was further diluted to 2 M urea and digested with trypsin (1:50, w/w) for 16-20 hours at 29 ℃.

(4) The digested peptide mixture was acidified with 10 % TFA to achieve a final concertation of 0.5 %.

(5) Peptide sample was desalted with a homemade StageTip packaged with SDB-XC membrane disk and C18-beads (10 mg beads per tip).

(6) The peptide concentration was determined using BCA assay.

3. Phosphopeptide enrichment

(1) Ni-NTA agarose beads were suspended with 6 % AA (pH 3.0) and loaded into a D200 micro-tip with a frit-disk capped in the end.

(2) The beads were deactivated with 50 mM EDTA in 1 M NaCl and equilibrated with 6 % AA.

(3) The beads were activated with 100 mM $FeCl_3$ in 6 % AA and equilibrated with 6 % AA.

(4) The peptide in 80% ACN contained 0.1 % TFA was loaded into the activated micro-tip.

(5) Sample loaded micro-tip was washed with 80 % ACN contained 0.1 % TFA and 1 % AA for twice.

(6) The phosphopeptides were eluted with 200 mM $NH_4H_2PO_4$ and desalted with homemade StageTip packed with two-layer C18 membrane disk.

(7) Phosphopeptide sample was dried with vacuum centrifugation and stored in -80 ℃ until LC-MS/MS analysis.

Experimental Protocol: MeOH-based protein extraction (ME and MU)

1. Protein extraction

(1) An equal weight of bacterial pellet was lysed with 50 % MeOH, either with 8 M urea (MU) or without urea (ME). The buffer volume used was 40 µL per 10 mg of pellet.

(2) The well-mixed sample was submitted for homogenization using Bioruptor Plus with frequency of 30s on/ 30s off per cycle, high energy mode, under 4 ℃. A total of 40 cycles were applied for Gram-positive bacteria, while 25 cycles were used for Gram-negative bacteria.

(3) The homogenized sample was subjected to MeOH/$CHCl_3$ protein precipitation. For ME, the sample was added with ½-fold its volume of MeOH and $CHCl_3$. Vertex vigorously to ensure well-mix and stand on-ice for 30 mins. (note: final ratio of MeOH : $CHCl_3$: H_2O = 2 : 1 : 1, v/v/v). For MU, the sample was added with ½-fold its volume of MeOH, ¼-fold its volume of $CHCl_3$, and ¼-fold its volume of H2O, followed by vertexing to ensure thorough mixing. (note: final ratio of MeOH : $CHCl_3$: H_2O = 2 : 1 : 2, v/v/v)

(4) The protein pellet was collected by centrifugation at 17,000 xg for 10 mins at RT and the white protein precipitate was allowed to air dry and dissolved with 8 M urea in 50 mM TEABC.

(5) The protein concentration was determined using BCA assay, the following manufacturer's guidelines.

2. In-solution protein digestion and peptide desalting

(1) Protein extract in 8 M urea/50 mM TEABC was reduced and alkylated at 29 ℃ by 10 mM TCEP for 30 mins and 40 mM CAA for 45 mins, respectively.

(2) The sample was diluted with 50 mM TEABC to a urea concentration of 4 M, followed by digestion with Lys-C (1:100, w/w) for 3 hrs at 29 ℃.

(3) The sample was further diluted to 2 M urea and digested with trypsin (1:50, w/w) for 16-20 hours at 29 ℃.

(4) The digested peptide mixture was acidified with 10 % TFA to achieve a final concertation of 0.5 %.

(5) Peptide sample was desalted with a homemade StageTip packaged with SDB-XC membrane disk and C18-beads (10 mg beads per tip).

(6) The peptide concentration was determined using BCA assay.

3. Phosphopeptide enrichment

(1) Ni-NTA agarose beads were suspended with 6 % AA (pH 3.0) and loaded into a D200 micro-tip with a frit-disk capped in the end.

(2) The beads were deactivated with 50 mM EDTA in 1 M NaCl and equilibrated with 6 % AA.

(3) The beads were activated with 100 mM $FeCl_3$ in 6 % AA and equilibrated with 6 % AA.

(4) The peptide in 80 % ACN contained 0.1 % TFA was loaded into the activated micro-tip.

(5) Sample loaded micro-tip was washed with 80 % ACN contained 0.1 % TFA and 1 % AA for twice.

(6) The phosphopeptides were eluted with 200 mM $NH_4H_2PO_4$ and desalted with homemade StageTip packed with two-layer C18 membrane disk.

(7) Phosphopeptide sample was dried with vacuum centrifugation and stored in -80 ℃ until LC-MS/MS analysis.
